# Comparison of O-Antigen Gene Clusters of All O-Serogroups of *Escherichia coli* and Proposal for Adopting a New Nomenclature for O-Typing

**DOI:** 10.1371/journal.pone.0147434

**Published:** 2016-01-29

**Authors:** Chitrita DebRoy, Pina M. Fratamico, Xianghe Yan, GianMarco Baranzoni, Yanhong Liu, David S. Needleman, Robert Tebbs, Catherine D. O'Connell, Adam Allred, Michelle Swimley, Michael Mwangi, Vivek Kapur, Juan A. Raygoza Garay, Elisabeth L. Roberts, Robab Katani

**Affiliations:** 1 *E*. *coli* Reference Center, Department of Veterinary and Biomedical Sciences, The Pennsylvania State University, University Park, Pennsylvania, United States of America; 2 Eastern Regional Research Center, Agricultural Research Service, U.S. Department of Agriculture, Wyndmoor, Pennsylvania, United States of America; 3 Animal Health & Food Safety, Life Sciences Solutions, Thermo Fisher Scientific, Austin, Texas, United States of America; University of Nottingham, UNITED KINGDOM

## Abstract

*Escherichia coli* strains are classified based on O-antigens that are components of the lipopolysaccharide (LPS) in the cell envelope. O-antigens are important virulence factors, targets of both the innate and adaptive immune system, and play a role in host-pathogen interactions. Because they are highly immunogenic and display antigenic specificity unique for each strain, O-antigens are the biomarkers for designating O-types. Immunologically, 185 O-serogroups and 11 OX-groups exist for classification. Conventional serotyping for O-typing entails agglutination reactions between the O-antigen and antisera generated against each O-group. The procedure is labor intensive, not always accurate, and exhibits equivocal results. In this report, we present the sequences of 71 O-antigen gene clusters (O-AGC) and a comparison of all 196 O- and OX-groups. Many of the designated O-types, applied for classification over several decades, exhibited similar nucleotide sequences of the O-AGCs and cross-reacted serologically. Some O-AGCs carried insertion sequences and others had only a few nucleotide differences between them. Thus, based on these findings, it is proposed that several of the *E*. *coli* O-groups may be merged. Knowledge of the O-AGC sequences facilitates the development of molecular diagnostic platforms that are rapid, accurate, and reliable that can replace conventional serotyping. Additionally, with the scientific knowledge presented, new frontiers in the discovery of biomarkers, understanding the roles of O-antigens in the innate and adaptive immune system and pathogenesis, the development of glycoconjugate vaccines, and other investigations, can be explored.

## Introduction

O-antigens are part of the lipopolysaccharide (LPS) on the outer envelope of *Escherichia coli*. LPS exhibits a tripartite structure, including the lipid A, core oligosaccharide, and the O-polysaccharides or O-antigens. The O-antigen domain is composed of repeating units of one or more sugar residues, exhibiting remarkable diversity in structure. Variation in the combination, position, stereochemistry, and links between these sugars and the presence or absence of non-carbohydrate entities makes them the most variable region in the cell [1, 2]. Since O-antigens that define the serogroups are important virulence factors and targets of both the innate and adaptive immune systems, their roles in both human and veterinary medicine have evoked considerable interest.

A method based on the identification of the combination of three principal cell surface components, the O-antigens, flagellar H-antigens, and capsular K-antigens was developed for subtyping *E*. *coli* strains. Since few laboratories had capabilities to type K-antigens, serotyping based on O- and H-antigens became the “gold standard” for *E*. *coli* typing. In the 1940s, Kaufmann [3–5] classified *E*. *coli* by serological methods, and by 1945 he successfully classified *E*. *coli* on the basis of the antigenic properties. Ørskov *et al*. [6] presented a comprehensive serotyping system for *E*. *coli* strains for 164 O-groups, which has been the basis for O-classification for taxonomic and epidemiological studies and for distinguishing strains during outbreaks and for surveillance.

O-groups O1-O187 have been defined, although O-groups O31, O47, O67, O72, O94 and O122 are no longer valid and have been withdrawn [7, 8], and four groups have been divided into subtypes: O18ab/ac, O28ab/ac, O112ab/ac and O125ab/ac, giving a total of 185 O-groups. In addition, there are 11 other OX-groups informally used by several laboratories (including ours), thus making 196 designated O-groups. Serotyping, the standard method for detecting the O-groups, is based on agglutination reactions of the O-antigen and antisera generated against each of the O-types. Serotyping is labor intensive and error-prone due to cross-reactivity between adsorbed O-antigen antisera produced in rabbits. Some strains are non-typeable, and others can be rough or autoagglutinating, making these cultures un-typeable.

Genes required for the biosynthesis of *E*. *coli* O-antigens are located on the chromosomal O-antigen gene cluster (O-AGC) flanked between a conserved 39-bp JUMPstart sequence (upstream), which is downstream of *galF* (UTP-glucose-1-phosphate uridylyltransferase) and *gnd* (6-phosphogluconate dehydrogenase) [9, 10]. The O-antigen biosynthesis genes in the O-AGC vary considerably for each serogroup. There are three mechanisms known for the processing of the O-antigen that generally consists of 10–25 repeating units of two to seven sugar residues. There is one mechanism that is O-antigen polymerase, Wzy dependent, where individual repeat units of O-polysaccharides are assembled at the cytoplasmic face of the inner membrane and are transported across the membrane by O-antigen flippase, Wzx. Polymerization of new units of polysaccharides occurs in the periplasmic face of the inner membrane by Wzy (O-antigen polymerase) and is typical for heteropolysaccharides. The majority of *E*. *coli* O-antigens are Wzx/Wzy-dependent. With the ABC-transporter-dependent pathway, typical for homopolymers, the extension of the O-antigen repeat unit occurs entirely on the cytoplasmic face of the inner membrane by glycosyl transferases followed by transport across the membrane by the ABC transporter system [11]. The third system is the synthase-dependent exopolysaccharide secretion system in which the glycosyl transferases are responsible for transport of the polysaccharide across the membrane; this system is not well comprehended. Although, key components of this pathway have recently been identified in *E*. *coli*, they only appear to function in the transport of specific exopolysaccharides [12].

In the last decade, significant progress has been made in identifying the *E*. *coli* O-groups by molecular methods, especially for serogroups associated with diseases in humans and animals. The sequences of the O-unit processing genes, the *wzx* (O-antigen flippase) and *wzy* (O- antigen polymerase) are relatively unique for each individual O-type. Therefore, these two genes were targeted for PCR assays and microarrays to identify the *E*. *coli* O-groups [13–17]. Lin *et al*. [18] combined PCR with the Luminex system to identify ten pathogenic Shiga toxin-producing *E*. *coli* O-groups. The amplified *wzx* and *wzy* targets were bound to fluorescent microspheres conjugated with complementary DNA probes in the Luminex system. Multiplex assays targeting several O-serogroup genes [15, 19] and virulence genes have been developed [20, 21]. While for Wzy-dependent O-AGCs, the PCR assays targeted the *wzx* and *wzy* genes, *wzm* and *wzt* genes have been targeted for the detection of for ABC transporter-dependent O-AGC, O8, O9, O52 and O101 [16, 22–24]. Microarrays for genoserotyping were designed for detecting O-groups, H-types, and virulence genes that allowed comprehensive typing of *E*. *coli* strains using the GeneAtlas system from Affymetrix [25, 26]. Other methods such as flow cytometry [27], immunoassays [28, 29] and microarrays using antibodies [30, 31] have also been developed for rapid detection of Shiga toxin-producing *E*. *coli* O-groups.

The objectives of this study were to compare the nucleotide sequences of all 196 O-AGCs of *E*. *coli* in conjunction with their serological reactions. The gene sequences of 71 O-AGCs were determined and submitted to GenBank and the comparative genetics of 196 O-AGCs of *E*. *coli* are presented with suggestions for updating the nomenclature for *E*. *coli* O-groups. This study may be leveraged to discover biomarkers for developing rapid, convenient, and accurate methods for O-group determination. The sequences could be potentially utilized to study the comparative evolution of O-antigens of bacteria that may occur through gene deletion, acquisition, or inactivation, mechanisms of host adaptation and immune system evasion, expression of virulence, and development of glycoconjugate vaccines for diseases, as well as for other purposes.

## Materials and Methods

### Bacterial strains and culture conditions

The reference control standard strains that were sequenced are used routinely for O-serotyping at the *E*. *coli* Reference Center at the Pennsylvania State University [6]. The strains were obtained from Statens Serum Institut (SSI) in Denmark that is affiliated with the World Health Organization Collaborating Centre for Reference and Research on *Escherichia* and *Klebsiella*. The strains are listed in S1 Table. All bacteria were grown in Luria Bertani (LB) broth or on LB agar plates at 37°C.

### Genome sequencing, assembly, and annotation

Genomic DNA was isolated using the PureLink Genomic DNA Mini kit (Thermo Fisher Scientific, Inc., Waltham, MA). The concentration of DNA was measured by absorbance readings at 260 nm and 280 nm using the Nanodrop ND100 UV-Vis spectrophotometer (Nanodrop Technologies, Wilmington, DE). DNA libraries for sequencing on the Ion Torrent Personal Genome Machine (PGM) (Thermo Fisher Scientific, Inc.) were prepared following the manufacturer's recommended library construction procedures. Ion Torrent PGM Ion 316 or 318 v2 chips with either the 200-bp or 400-bp OneTouch kits were used for generating sequence data. The *de novo* assembly of whole genomes into the final contigs was performed with CLC Genomics Workbench 7.0 (CLC Bio, Aarhus, Denmark) using the default settings. The published primers complementary to JUMPStart and *gnd* [32] were mapped to the final contigs with a minimum sequence identity of 70% over a window of 20 nucleotides. When necessary joining of O-AGC contigs was performed by using Sanger sequencing and joining of long PCR amplicons as described [32]. GeneWise [33] was used to predict gene structure and check for frameshifts and sequencing errors. In addition, Prokka 1.10 software [34] in combination with manual annotation was used to finalize the gene structure of the O-AGCs before submission to GenBank. The HMMTOP 2.0 transmembrane topology prediction server [35] was used to identify potential transmembrane helices from the amino acid sequences.

### Construction of the phylogenetic tree

A phylogenetic tree of the 196 O-AGCs was generated using the DNA sequences between the JUMPstart and GND primers. Both the alignment and the phylogenetic tree were generated using CLC Genomics Workbench 8.5.1. To create the alignment, the following parameters were selected: Gap open cost = 10.0, Gap extension cost = 1.0, and selecting the very accurate progressive alignment. To create the phylogenetic tree, the Maximum Likelihood Phylogeny tool was selected and analysis was performed under the assumption of the Jukes Cantor substitution model within the software program. The Neighbor Joining construction method was selected. To determine the reliability of the tree, 100 bootstrap replicates were performed.

### GenBank accession numbers

All O-antigen cluster sequences were deposited in the NCBI GenBank database and the accession numbers are listed in S1 Table.

## Results and Discussion

### Structure of the O-AGCs

**Fig 1:**
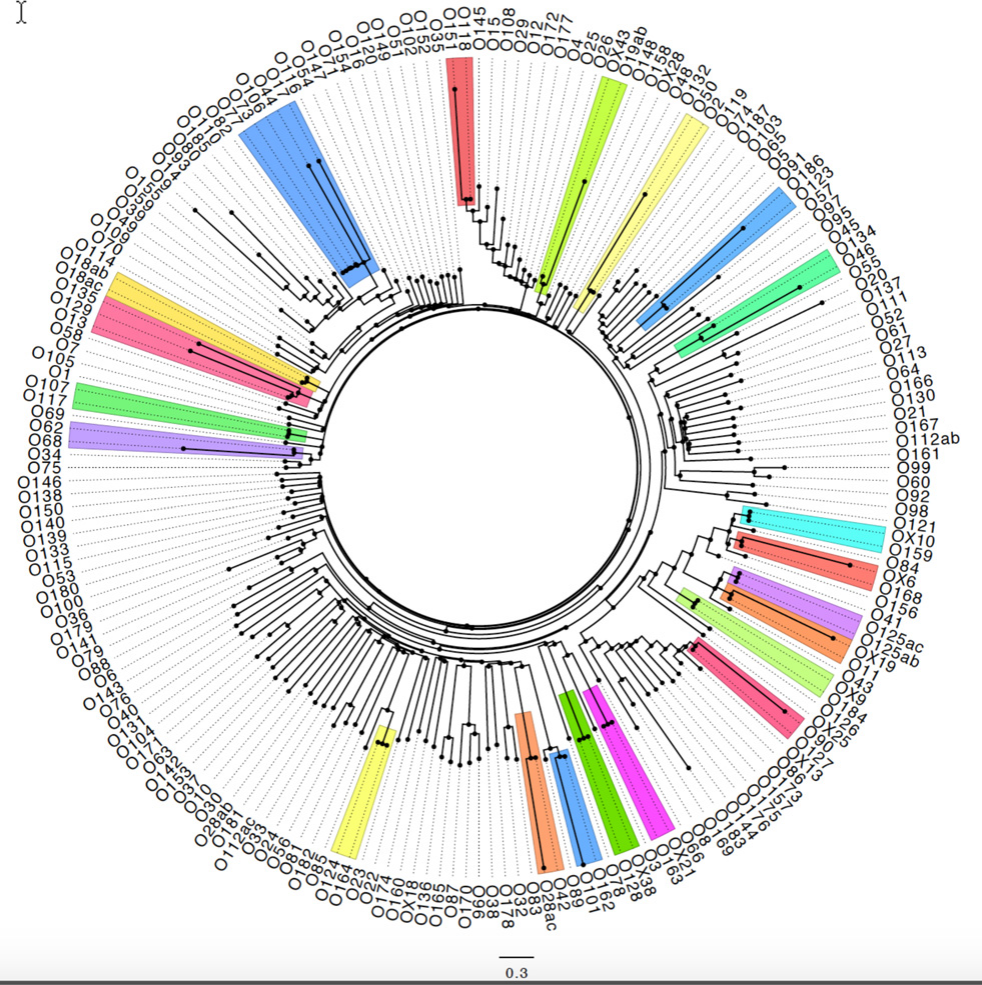
Phylogenetic tree for all O-AGCs of *E*. *coli*. The O-AGCs that show 98–99.9% relatedness are highlighted.

To characterize the genetic diversity of the O-AGCs, the DNA sequences generated either from the current study or from nucleotide sequences published in GenBank (Accession numbers are listed in S1 Table), including insertion elements, and other non-coding regions between the JUMPstart and *gnd* regions from 196 O-AGCs were compared using the maximum likelihood phylogenetic tool of the CLC Genomics Workbench. The comparative phylogenetic tree is depicted in Fig 1. Since insertion elements play an important role in the evolution of O-AGCs, these were included to present a more complete comparison of the relationship among the clusters [32, 36]. The number of genes in the O-AGC varied between five (O174) and 18 (O108) and the lengths ranged from 5.6 kb (O174) to 27.7 kb (O55) (S1 Fig). The genes encoding for the O-antigens belong to three major categories. The nucleotide sugar biosynthesis genes that are involved in the synthesis of O-antigen nucleotide sugar precursors, the glycosyl transferases, that transfer the various sugar precursors to form the oligosaccharide, and the O-antigen processing proteins, the flippase (Wzx), O-antigen polymerase (Wzy) and polysaccharide ABC transporter, O-antigen ABC transporter permease Wzm, and O-antigen ABC transporter ATP-binding protein Wzt.

Serogroups, O14 and O57, do not carry O-AGC-related genes between *galF* and *gnd* loci, and therefore, could not be mapped. Serogroup O14 is known to be rough and cannot be serotyped [6], and has been previously reported to lack an O-AGC [37, 38]. Antisera raised against O14:K7 (a rough strain) have been shown to cross-react against *E*. *coli* and other Enterobacteriaeceae due to the presence of the enterobacterial common antigen to which the antisera react [38]. Similarly, other investigators could not locate an O-AGC in O57 [37, 39].

### Nucleotide sugar biosynthesis genes

Nucleotide sugar biosynthesis genes exhibit a high level of identity among the different O-groups and often group together in the cluster. A notable number of these genes are conserved in various species. There are four genes, *rmlB* (dTDP-glucose 4, 6-dehydratase), *rmlD* (dTDP-4-dehydrorhamnose reductase), *rmlA* (glucose-1-phosphate thymidylyltransferase), and *rmlC* (dTDP-4-dehydrorhamnose 3,5-epimerase) that are involved in the biosynthesis of dTDP-L-rhamnose. In 49 O-AGCs, these are grouped as *rmlBDAC* (S1 Fig). In 30 O-AGCs, part of the group is separated or missing. In O2, O50, O54, O62, O71, O109, O119 and O177, *rmlC* is separated from the group due to insertion of other genes between *rmlA* and *rmlC*, and in others, only two of the genes in the group such as *rmlDA* or *rmlBA* are present. The *manB* gene encoding for phosphomannomutase and *manC* encoding for mannose-1-phosphate guanyltransferase responsible for the biosynthesis of GDP-D-mannose [40] are present in 56 O-AGCs. The two genes involved in biosynthesis of UDP-L-FucNAc derived from UDP-GlcNAc, *fnlA* (UDP-glucose epimerase) and *fnlC* (UDP-N-acetylglucosamine 2-epimerase), were identified in 15 O-AGCs. VioA and VioB that carry out transamination of dTDP-6-deoxy-D-xylo-4-hexulose to dTDP-4-amino-4,6-dideoxy-D-glucose (VioN) and VioB that N-acetylates VioN to dTDP-VioNAc were found to be associated with the O-AGC for O39, O49, and O116.

### Glycosyl transferases

Glycosyl transferases are responsible for adding sugar residues to the O-antigens during their synthesis. Numerous combinations of an extensive range of sugars are present in O-antigens, with specific linkages among them. Therefore, heterogeneous groups of highly specific glycosyl transferases are associated with the O-AGCs. These were identified based on sequence similarities to other sugar transferases that are found within the O-AGCs.

### O-antigen processing genes

The O-antigen processing genes, *wzx* (flippase) and *wzy* (polymerase), are highly specific for each O-group and are present in most of the O-AGCs. The O-antigen is synthesized when a glycosyl-1-phosphoryl residue is transferred to an undecaprenyl phosphate acceptor to form an undecaprenyl-PP-sugar intermediate. Transfer of additional sugar units to this undecaprenyl results in an undecaprenyl-PP-oligosaccharide intermediate to which repeating sugar units are sequentially transferred, and are then translocated and flipped across the membrane by Wzx [40]. Both Wzx and Wzy are hydrophobic proteins with transmembrane helices, and they show high variation in sequence. These genes are involved in the synthesis and translocation of O-antigens using the Wzy-dependent pathway. The O-AGCs of 185 O-groups carry the O-antigen flippase (*wzx*) and O-antigen polymerase (*wzy*) genes, as confirmed in our analyses. Eleven O-AGCs: O8, O9, O52, O60, O89, O92, O95, O97, O99, O101 and O162 are ABC transporter-dependent for O-antigen processing and carry *wzm* and *wzt* that assist in the transport process. The mechanisms of O-antigen biosynthesis in O8 and O9, that have capsules, have been extensively studied [11]. Although O8 has *wzx* and *wzy* genes in the O-AGC, the genes in the cluster are directed to form a capsule and the O-polysaccharides are transported using an ATP-binding ABC-transporter process [11, 24, 40, 41]. The O-AGCs of O89, O101 and O162 are notably identical as discussed later and therefore, there are nine unique O-AGCs that are ABC transporter-dependent.

### Relatedness among O-AGCs

**Fig 2:**
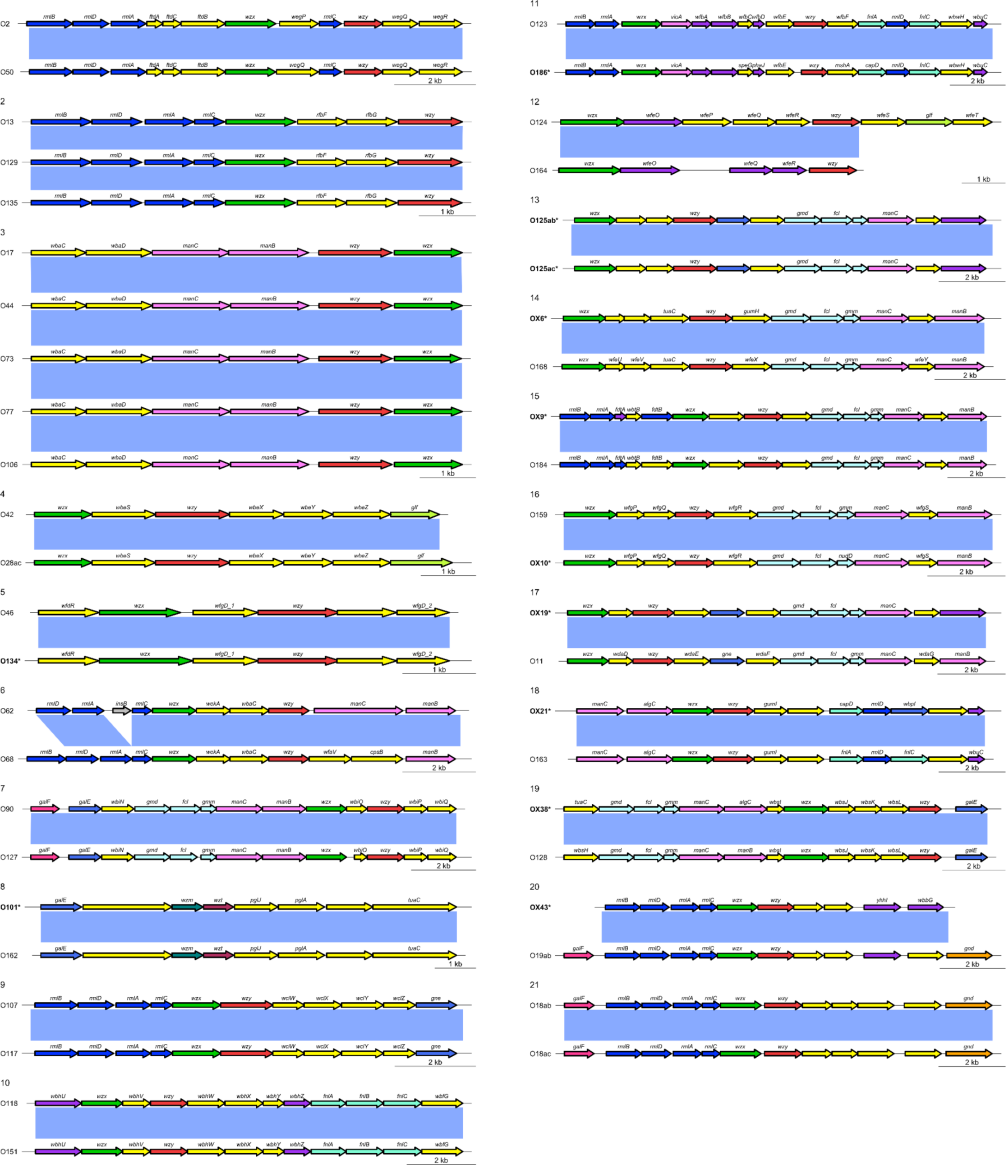
Comparison of identical O-AGCs.

Analysis of the phylogenetic relatedness among 196 O-AGCs demonstrated that twenty sets of O-groups were 98–99.9% identical in their nucleotide sequences as highlighted in Fig 1. Diagrammatic representations of the genes representing these 21 sets of identical O-AGCs, are presented in Fig 2. These are O2/O50, O13/O129/O135, O17/O44/O73/O77/O106, O42/O28ac, O46/O134, O62/O68, O90/O127, O101/O162, O107/O117, O118/O151, O123/O186, O124/O164, O125ab/O125ac, OX6/O168, OX9/O184, OX10/O159, OX19/O11, OX21/O163, OX38/O128, OX43/O19, O118ab/O118ac. The O-AGCs of O62 and O68 differ due to the presence of an insertion element located within the third codon from the end of the *rmlA* gene in O62 otherwise, they are almost identical [32] (Fig 1). As mentioned above, insertion elements play a role in the evolution of O-AGCs.

The comparative serological cross-reactivity data for these sets of identical O-groups, as observed and recorded for the last 50 years of serotyping at the *E*. *coli* Reference Center are listed in Table 1. Although the nucleotide sequences may be identical in certain O-groups, the serological reactions with rabbit antisera may not show any cross-reactivity as observed for strains belonging to O2/O50, O46/O134, O118/O151, and OX19/O11. This could be due to post-translational modification of proteins that may be responsible for the epitopes in antigens. Recently Joensen *et al*. [42] presented information on cross-reactions of the O-groups that have 98–100% identical *wzx* and *wzy* genes. Although there are some differences in cross-reactions they observed between identical O-groups that are different from ours, some are similar. For example, serogroups O107 and O117 and serogroups O123 and O186, show serological cross-reactivity in both studies; however, Joensen and co-workers [42] stated that serogroups O2 and O50 cross-reacted serologically, while in the current study no cross-reaction was observed. Cross-reactions between O-groups vary considerably, and may depend on the polyclonal antisera generated in different rabbits. Further research may elucidate the mechanism of antigen-antibody reactions for these O-groups. Some of the O-groups such as O90 sometimes cross-react with O127 but not vice versa; O101 may sometimes cross-react with O162, but O162 does not cross-react with O101, and the reason for this is unclear. Many of the genetically similar O-groups that are related do cross-react as shown in Table 1. Strains that react serologically with O17 antisera were sometimes found to cross-react with antisera generated against O73, O77 and O106 but never with O44, suggesting that the epitopes for the immunologic reactions may vary based on the whole genome composition. O-AGCs of O118 and O151 exhibit identical nucleotide sequences, except O151 carries substitutions in two nucleotides thereby altering two amino acids in the proteins that are translated [43], they do not cross-react serologically.

**Table 1:** Comparison of O-AGC of O-groups that are 98–99.9% identical.

Set #	Computational analysis	Serological cross-reaction	Comparison Status of sequences	Availability^a^
1	O2/O50	No	*in silico*	O2 (*n* = 2650)/ O50 (*n* = 46)
2	O13/O129/O135	Yes	*in silico*	O13 (*n* = 280)/ O129 (*n* = 42)/ O135 (*n* = 45)
3	O17/O44 /O73/ O77/O106	Yes	*in silico*	O17 (*n* = 322)/ O44 (*n* = 109)/ O73 (*n* = 604)/ O77 (*n* = 253)/ O106 (*n* = 229)
4	O42/O28ac	Insufficient data	*in silico*	O42 (*n* = 41)/O28 (*n* = 42)
5	O46/O134	No	This study	O46 (*n* = 60)/O134 (*n* = 28)
6	O62/O68	O68 cross-reacts with O62 but not vice versa	Liu Y et al. 2015	O62 (*n* = 2)/O68 (*n* = 127)
7	O90/O127	O90 cross-reacts with O127 but not vice versa	*in silico*	O90 (*n* = 25)/O127 (*n* = 51)
8	O101 /O162	O101 cross-react with O162 but not vice versa	*in silico*	O101 (*n* = 734)/O162 (*n* = 98)
9	O107/O117	Yes	*in silico*	O107 (*n* = 59)/O117 (*n* = 239)
10	O118/ O151	No	*in silico*	O118 (*n* = 182)/O151 (*n* = 61)
11	O123/O186	Yes	*in silico*	O123 (n = 45)/O186 (n = 4)
12	O124/ O164	Occasional cross-reaction	*in silico*	O124 (*n* = 91)/O164 (*n* = 11)
13	O125ab/O125ac	Insufficient data	This study	O125 (*n* = 54)
14	OX6/ O168	Yes	This study	OX6 (*n =* 46)/O168 ((*n =* 99)
15	OX9/O184	Insufficient data	This study	OX9 (*n =* 124)/O184 (*n =* 1)
16	OX10/O159	Yes	This study	OX10 (*n =* 81)/O159 (*n =* 161)
17	OX19/O11	No	This study	OX19 (*n =* 28)/O11 (*n =* 527)
18	OX21/O163	Yes	This study	OX21 (*n =* 26)/O163 (*n =* 137)
19	OX38/O128	Insufficient data	This study	OX38 (*n = 15*)/O128 (*n =* 329)
20	OX43/O19	Yes	This study	OX43 (*n =* 40)/O19 (*n = 385*)
21	O18ab/O18ac	Insufficient Data	*in silico*	O18 (n = 777)

a, Number of cultures in database of *E*. *coli* Reference Center (ECRC) (1967–2015)

**Fig 3:**
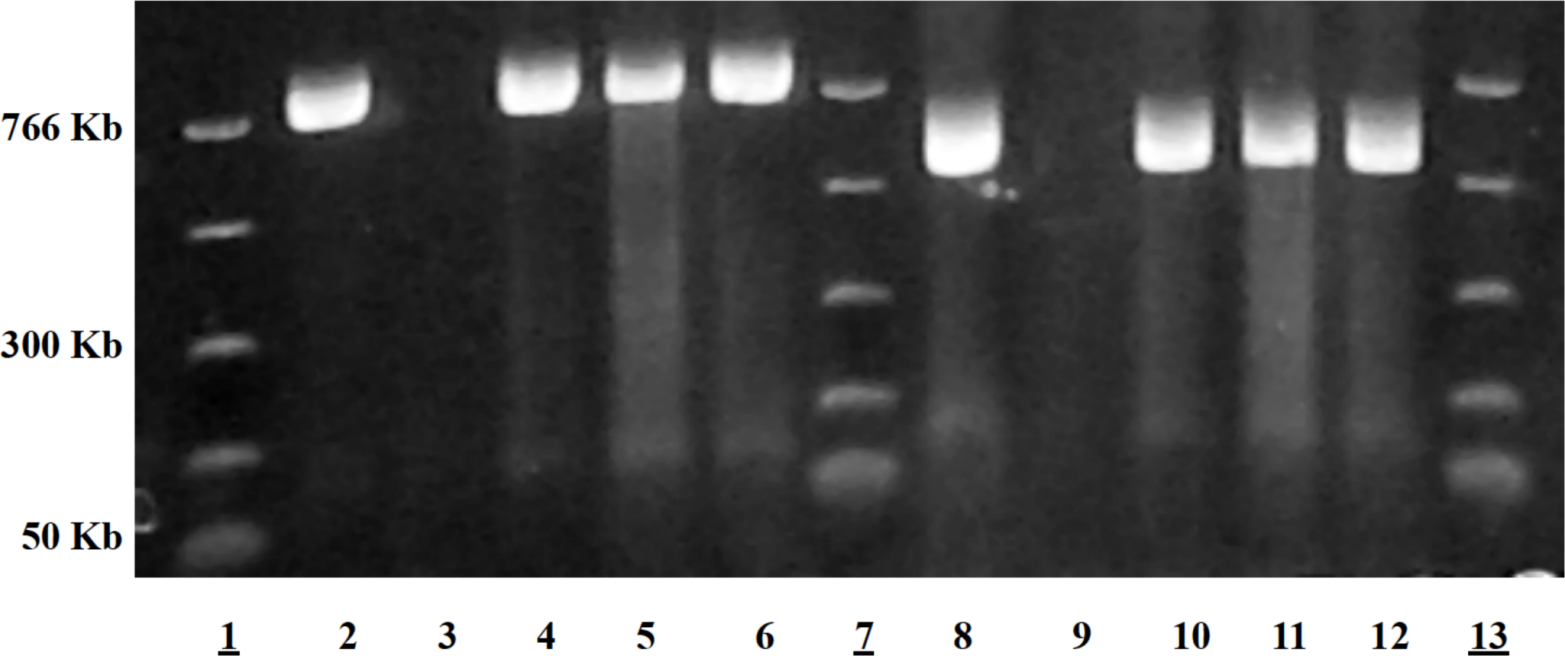
O153 *wzx* and *wzy* genes amplified by PCR using primers from sequences presented in this investigation. Lanes 1, 7, 13: Molecular weight markers. Lane 2: Positive control for O153 targeting *wzx* gene, Lane 3: Negative control, Lanes 4,5,6: *wzx* amplified for three clinical isolates. Lane 9: Positive control for O153 targeting *wzy* gene. Lane 10: Negative control, Lane 11,12,13: *wzy* amplified for three clinical isolates.

Iguchi *et al*. [37] assigned O-AGCs of all 184 O-groups of *E*. *coli* into 16 groups based on similarities in nucleotide sequences. Most of the groups they describe match with our results except for O153 and O137 [37]. No significant similarities in nucleotides sequences were observed for O153/O178 in the current study (Fig 1, S1 Fig). PCR assays developed targeting *wzx* and *wzy* genes from GenBank sequences submitted in this report (KJ755551) for O153 were highly specific for clinical isolates belonging to serogroup O153 (Fig 3). Therefore, grouping O-AGCs O153 and O178 based on 99.9% identity may not be accurate [37]. Similarly, O-group O137, reported to be 99.7% identical to O20 [37], was not corroborated in the present investigation. The sequence of O137 (KJ755548) generated in the current study matches 100% with the nucleotide sequence published earlier for this O-group [44] (GenBank accession number GU068043) and is not identical to O20 (S1 Fig). While O89, O101, and O162 were grouped based on nucleotide similarities [37], our data show that O89 shares 96.6% identity over 66.6% coverage to O101 and O162, which are 99% identical over 100% coverage as determined by BLASTn [45] (Fig 1). Serogroup O89 is also serologically distinct from O101 and O162. Therefore, we believe O89 to be a distinct O-group. The O-AGCs of O169 and O183 were found to be 97% identical over 64% coverage (Fig 1), and thus are only partially similar. No serological cross-reaction between O169 and O183 was observed, and therefore, they could be considered as distinct O-groups.

The sequencing data generated will assist in developing platforms for molecular genoserotyping of *E*. *coli*. In order to develop the scheme, there is a need to consider merging or eliminating the designations of O-groups that have identical O-AGCs. Since O-AGCs for O14 and O57 could not be identified in their genomes, it will be difficult to designate these O-groups until target genes that may potentially be involved in the synthesis of O-AGCs are identified for these O-groups. Whole genome sequencing and gene expression studies with knock-out mutants for rough strains may also elucidate the complexity involving O-antigen synthesis for O14. It should be considered that the serogroups that are similar in nucleotide sequence and cross-react serologically (Table 2) may be merged to eliminate redundancy. O125ab/O125ac may be designated O125 and O18ab/O18ac may be merged as O18 as these O-groups have been found to be identical [37]. O19ab can be designated as O19. The carbohydrate structures of O13/O129/O135 have been found to be similar and related to *Shigella flexneri* [46]. The sequence of serogroup O13 is 99% identical with 100% coverage to O129 it is 99% identical with 82% coverage for O135. Strains belonging to these serogroups cross-react serologically; therefore, O13 may be merged with O129 and O135 and the merged O-groups designated as O13. O28ac and O42 are identical except for three point mutations exhibited in O42 in the *wbeX* and *wbeY* genes [21], and these serogroups cross-react. Thus, these may be merged and designated as O42. O107 and O117 may be merged as O107. O17/O44/O73/O77/O106 have identical nucleotide sequences and share a common four-sugar backbone O-subunit structure with each other and *Salmonella enterica* serogroup O:6,14 (H) [47]. All of these O-groups except *E*. *coli* O77 O-antigen, have substitutions of one or two glucose side branches at various positions in the O-unit backbone and cross-react with each other except for O44. Three genes were identified in the *E*. *coli* O44 genome within a putative prophage that are presumably involved in the glucosylation of the basic tetrasaccharide unit [47]. This may be the reason why O44 strains never serologically cross-react with the others in the group (O17/O73/O77/O106). Since the antigenic specificities for these O-groups are quite distinct, further investigations need to be conducted to determine if these O-groups can be merged. However, for genoserotyping assays these O-groups may not be distinguishable.

**Table 2:** O-groups that may be potentially merged based on similarities in O-AGC nucleotide sequence and serological cross-reactions.

Set #	Identity 98–99.9%	Suggested O group
1	O42/O28ac	O42
2	O13/O129/O135	O13
3	O107/O117	O107
4	O123/O186	O123
5	O125ab/O125ac	O125
6	O18ab/O18ac	O18
7	OX6/O168	O168
8	OX10/O159	O159
9	OX21/O163	O163
10	OX38/O128	O128
11	OX43/O19	O19

Eight O-groups have been previously designated as OX1-OX8 by Ewing *et al* [48]. OX1 is now designated as O170, OX2 as O169, and OX3 as O174. OX4 and OX6 were found to be similar to O146 and O171, respectively, OX5 is now designated as O168, and OX7 as O175 [8, 49]. In this investigation, many of the OX-groups were found to be identical to established groups and may be eliminated. OX6 can be designated as O171, OX10 as O159, OX21 as O163, OX38 as O128, and OX43 as O19. The other OX groups, including OX13, OX18, OX25, OX28, and OX38, were found to have unique O-AGCs. Although additional studies are needed, we propose that these OX-groups may be designated as new O-groups chronologically following the designation of the Statens Serum Institut that have now listed O188 serogroups. (http://www.ssi.dk/English/SSI%20Diagnostica/Products%20from%20SSI%20Diagnostica/Bacterial%20strains/E%20coli.aspx). It is likely that more O-groups will be discovered, as the nucleotide sequences of the large number of non-typeable strains may exhibit unique sequences that cannot be designated as any of the established O-groups [50]. We may be able to assign O-groups to non-typeable strains based on the genoserotyping as they may exhibit SNPs or mutations in the O-AGCs hampering the serological reaction, resulting in their designation as non-serotypeable [51]. Whole genome sequencing of the strains may reveal factors responsible for synthesis of the antigenic domains of the O-antigens.

Based on the nucleotide sequences of the O-AGCs, genoserotyping can be achieved by targeting the unique sequences for each O-group. While *wzx* and *wzy* are suitable targets for most of the O-groups, and among the O-groups that do not carry *wzx* and *wzy* genes, unique regions within the *wzm* and *wzt* genes could be utilized for detecting O-groups O8, O9, O52, O60, O89/O101/O162, O92, O95, and O97. Joensen *et al*. [42] recently presented serotyping based on *in silico* whole genome sequences. The publicly available web tool, SerotypeFinder hosted by the Center for Genomic Epidemiology (www.genomicepidemiology.org) is available for O- genoserotyping. The O-antigen genes *wzx*, *wzy*, *wzm*, and *wzt* and flagellin genes can be detected easily based on sequence data, and thus, this tool can be an alternate faster and cheaper method than serotyping. Other methods are also likely to develop from the information presented that may lead to more accurate and rapid O-typing of *E*. *coli*.

## Supporting Information

S1 Fig Structure of O-AGC of all 196 O-serogroups. The O-AGCs of all 196 O- and OX-groups are diagrammatically represented. The nucleotide sequences of 71 O-groups marked with asterisk and in bold font were determined in the present investigation.
(PDF)

S1 Table Strains and GenBank accession numbers for O-AGCs of all known *E*. *coli* O-groups. (DOCX)
