# Correction: Comparison of O-Antigen Gene Clusters of All O-Serogroups of *Escherichia coli* and Proposal for Adopting a New Nomenclature for O-Typing

**DOI:** 10.1371/journal.pone.0154551

**Published:** 2016-04-27

**Authors:** Chitrita DebRoy, Pina M. Fratamico, Xianghe Yan, GianMarco Baranzoni, Yanhong Liu, David S. Needleman, Robert Tebbs, Catherine D. O'Connell, Adam Allred, Michelle Swimley, Michael Mwangi, Vivek Kapur, Juan A. Raygoza Garay, Elisabeth L. Roberts, Robab Katani

The legend for S1 Fig is missing. Please view a corrected version of S1 Fig below.

## Supporting Information

S1 Fig Structure of O-AGC of all 196 O-serogroups. The O-AGCs of all 196 O- and OX-groups are diagrammatically represented. The nucleotide sequences of 71 O-groups marked with asterisk and in bold font were determined in the present investigation.
(PDF)
