# Supplementary figures and images for: Comparison of O-Antigen Gene Clusters of All O-Serogroups of Escherichia coli and Proposal for Adopting a New Nomenclature for O-Typing
Source: PLoS One. 2016 Jan 29;11(1):e0147434. doi: 10.1371/journal.pone.0147434 (PMC4732683; doi:10.1371/journal.pone.0147434)

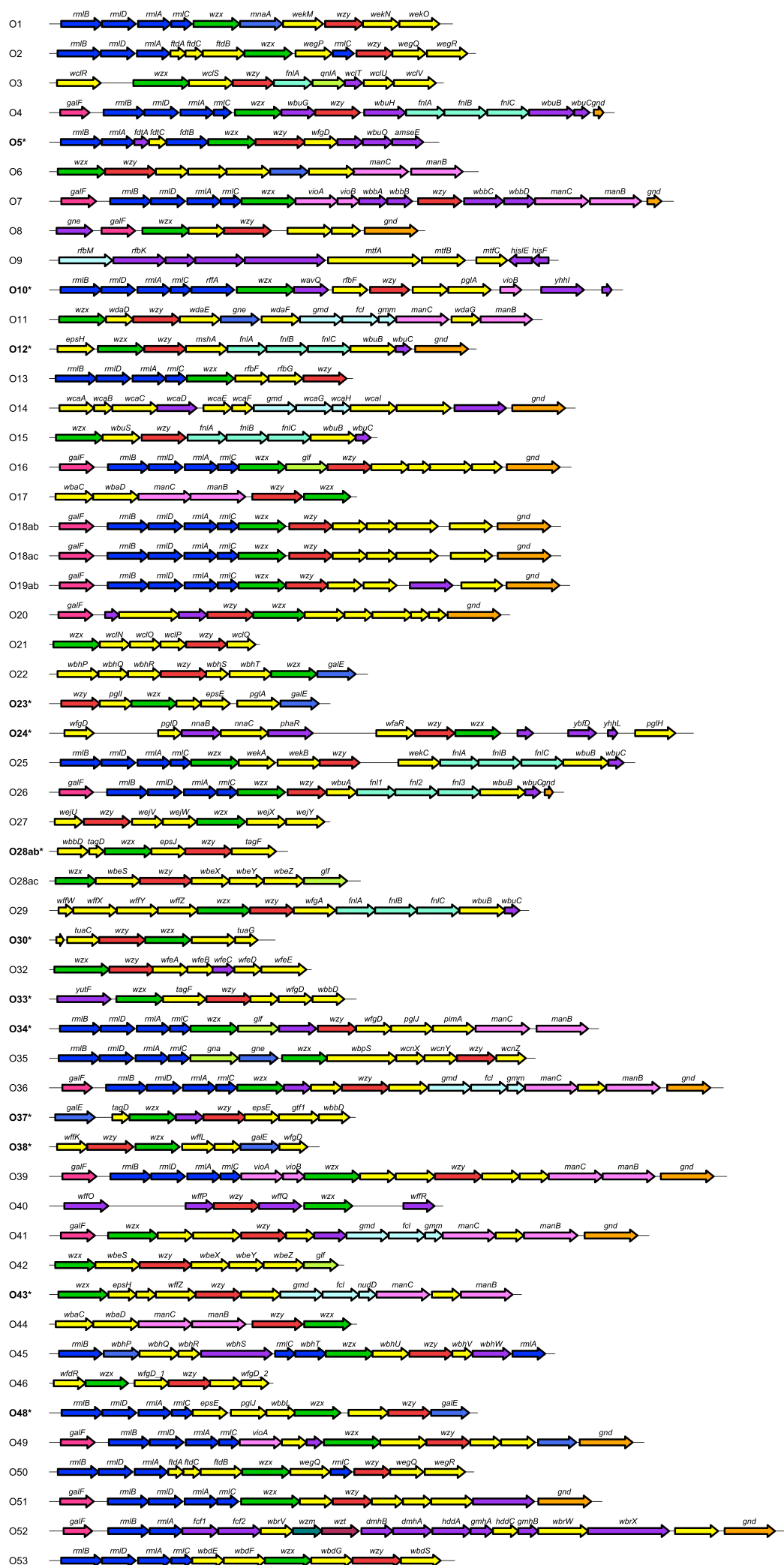

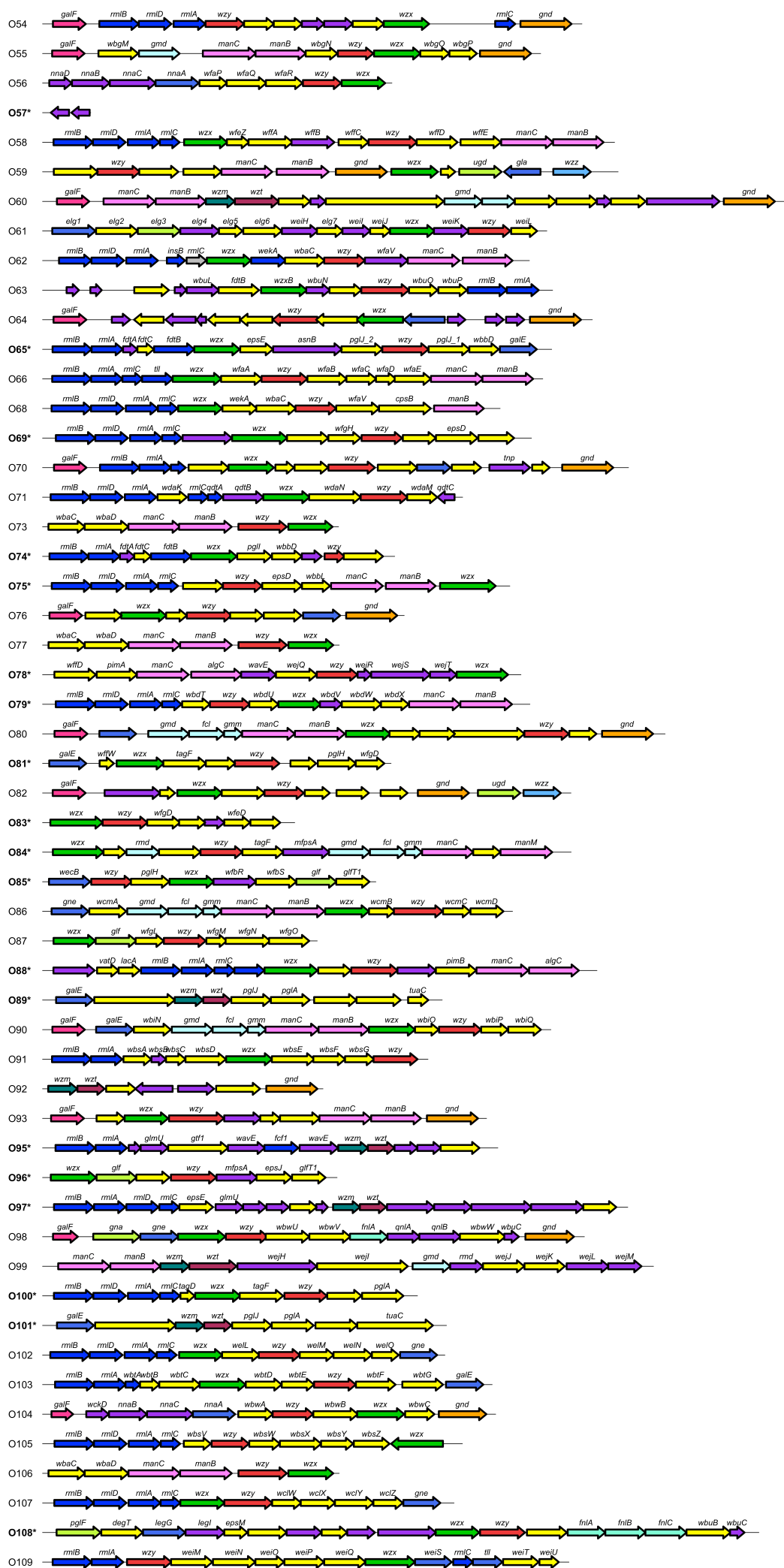



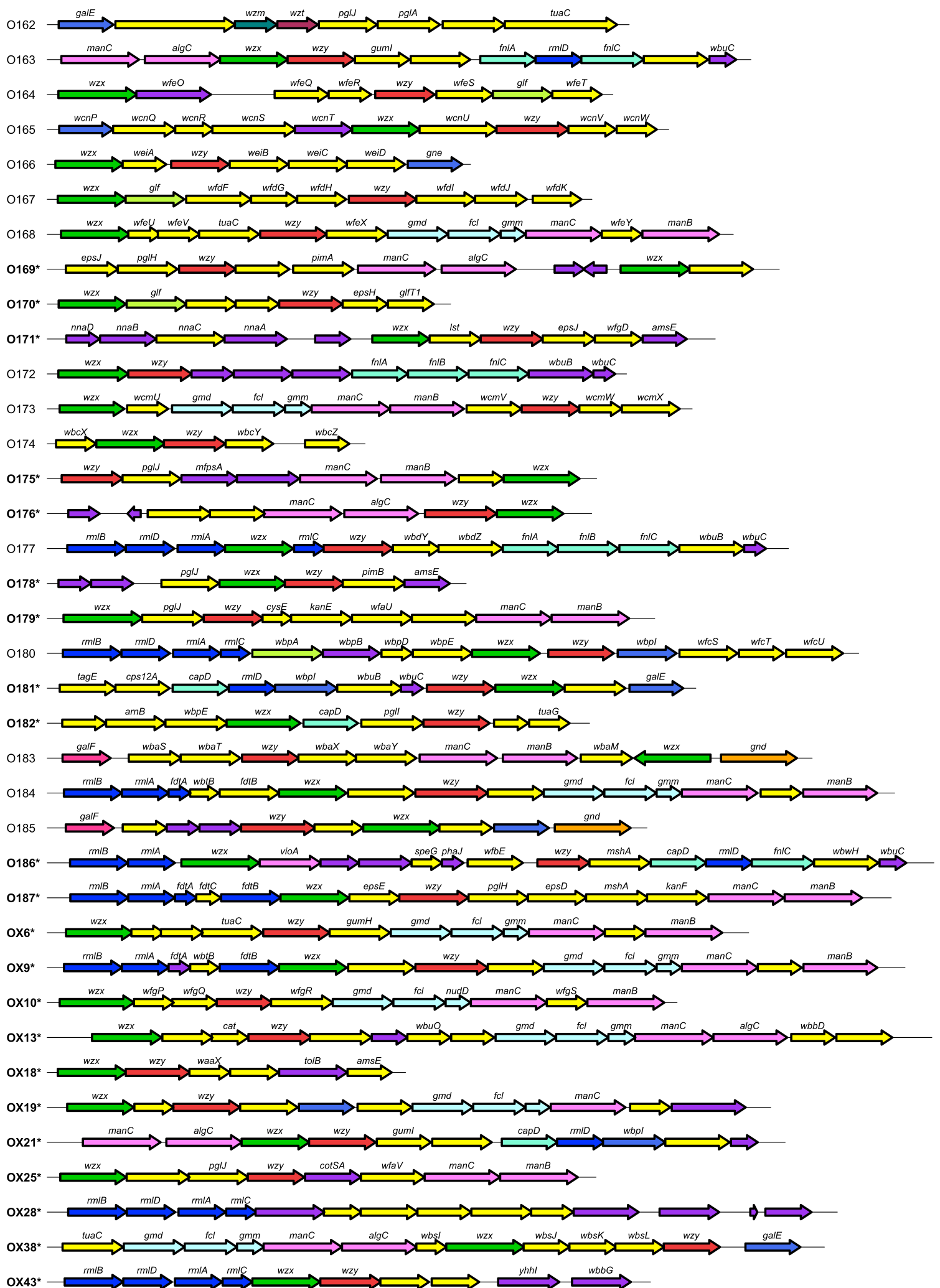

Supplement: S1 Fig — The O-AGCs of all 196 O- and OX-groups are diagrammatically represented. The nucleotide sequences of 71 O-groups marked with asterisk and in bold font were determined in the present investigation. (PDF) [file pone.0147434.s001.pdf]
