# Supplementary material for: Comparison of O-Antigen Gene Clusters of All O-Serogroups of Escherichia coli and Proposal for Adopting a New Nomenclature for O-Typing
Source: PLoS One. 2016 Jan 29;11(1):e0147434. doi: 10.1371/journal.pone.0147434 (PMC4732683; doi:10.1371/journal.pone.0147434)
Supplement: S1 Table — (DOCX) [file pone.0147434.s002.docx]

**S1 Table. Strains and GenBank accession numbers for O-AGCs of all known *E. coli* O-groups**

| **Serial #** | **O Type** | **Serotype** | **Strain #** | **GenBank Accession #** | **Reference** |
| --- | --- | --- | --- | --- | --- |
| 1 | O1 | O1 | G1632 | GU299791 | [1] |
| 2 | O2 | O2:H4 | U9-41 | EU549863 | [2] |
| 3 | O3 | O3 | - | EU694097 | [3] |
| 4 | O4 | O4 | - | AY568960 | [4] |
| 5 | O5 | O5:K4:H4 | U1-41 | KP710588 | This study |
| 6 | O6 | O6:K5:H1 | Nissel1917 | AJ426045 | [5] |
| 7 | O7 | O7:K1 | VW 187 | AF125322 | [6] |
| 8 | O8 | O8:K8:H4 | G3404-41 | AB811598 | [7] |
| 9 | O9 | O9:K31-:H- | F719 | D43637 | [8] |
| 10 | O10 | O10:K5:H4 | Bi8337-41 | KJ755557 | This study |
| 11 | O11 | O11 | G1207 | HQ388393 | [9] |
| 12 | O12 | O12:K5:H- | Bi626-42 | KJ755558 | This study |
| 13 | O13 | O13:K11:H11 | Su4321-41 | EU296422 | [10] |
| 14 | O14 | O14:K7:H- | Su4411-41 | AB972414.1 | [7] |
| 15 | O15 | O15:K2:H1 | F7902-41 | AY647261 | [11] |
| 16 | O16 | O16 | G1459 | HQ388392 | [9] |
| 17 | O17 | O17 | K12a | AB812084 | [7] |
| 18 | O18ab | O18 | G1630 | GU299793 | [1] |
| 19 | O18ac | O18 | D-M3219-54 | AB811603 | [7] |
| 20 | O19ab | O19ab:K- | F8188-91 | AB811604 | [7] |
| 21 | O20 | O20:K17:H- | P7a | AB811605 | [7] |
| 22 | O21 | O21 | - | EU694098 | [3] |
| 23 | O22 | O22:K13:H1 | E14a | DQ851855 | [12] |
| 24 | O23 | O23:K18:H15 | E39a | KJ755561 | This study |
| 25 | O24 | O24:K+:H- | E41a | KJ755562 | This study |
| 26 | O25 | O25:K19:H12 | E47a | GU014554 | [13] |
| 27 | O26 | O26:H- | 311b | AF529080 | [14] |
| 28 | O27 | O27:K-:H- | F9884-41 | GU014555 | [13] |
| 29 | O28ab | O28ab:K-:H- | K1a | KP710590 | This study |
| 30 | O28ac | O28ac:H25 | 96-3286 | DQ462205 | [2] |
| 31 | O29 | O29 | - | EU294173 | [10] |
| 32 | O30 | O30:K-:H- | P2a | KJ755563 | This study |
| 33 | O32 | O32 | - | EU296410 | [10] |
| 34 | O33 | O33:K-:H- | E40 | KJ755564 | This study |
| 35 | O34 | O34:K-:H10 | H304 | KJ778803 | This study |
| 36 | O35 | O35 | - | FJ940774 | [15] |
| 37 | O36 | O36:K-:H9 | H502a | AB811613 | [7] |
| 38 | O37 | O37:K-:H10 | H510c | KJ755554 | This study |
| 39 | O38 | O38:K-:H26 | F11621-41 | KP710589 | This study |
| 40 | O39 | O39:K-:H- | H7 | AB811616.1 | [7] |
| 41 | O40 | O40 | - | EU296417 | [10] |
| 42 | O41 | O41:K-:H40 | H710c | AB811617 | [7] |
| 43 | O42 | O42:K-:H37 | P11a | FJ539194 | [2] |
| 44 | O43 | O43:K-:H2 | Bi7455-41 | KJ778789 | This study |
| 45 | O44 | O44:H18 | H702c | AB811620 | [7] |
| 46 | O45 | O45:K1:H10 | H61 | AY771223 | [16] |
| 47 | O46 | O46:K-:H16 | P1c | AB811621 | [7] |
| 48 | O48 | O48:K-:H- | U8-41 | KJ710508 | This study |
| 49 | O49 | O49:K+:H12 | U12-41 | AB811623 | [7] |
| 50 | O50 | O50:K-:H4 | U18-41 | AB811624 | [7] |
| 51 | O51 | O51:K-:H24 | U19-41 | AB812020 | [7] |
| 52 | O52 | O52:K-:H10 | U20-41 | AY528413 | [17] |
| 53 | O53 | O53 | - | EU289392 | [10] |
| 54 | O54 | O54:K-:H2 | Su 3972-41 | AB812085 | [7] |
| 55 | O55 | O55:H7 | TB182 | AF461121 | [18] |
| 56 | O56 | O56:K+:H- | Su3684-41 | DQ220293 | [19] |
| 57 | O57 | O57:K-:H- | F8198-41 | KJ778813 | This study |
| 58 | O58 | O58 | - | EU294175 | [10] |
| 59 | O59 | O59:H19 | G1070 | AY654590 | [20] |
| 60 | O60 | O60 | F10167a-41 | AB812022 | [7] |
| 61 | O61 | O61 | - | GU220362 | [21] |
| 62 | O62 | O62:K-:H30 | F10524-41 | JX501334 | [22] |
| 63 | O63 | O63:K-:H- | F10598-41 | EU549862 | Fratamico and Yan 2008, unpublished |
| 64 | O64 | O64:K-:H- | K6b | AB812025 | [7] |
| 65 | O65 | O65:K-:H- | K11a | KP710592 | This study |
| 66 | O66 | O66 | - | DQ069297 | [23] |
| 67 | O68 | O68:K-:H4 | P7d | KJ534585 | This study |
| 68 | O69 | O69:K-:H38 | P9b | KJ778804 | This study |
| 69 | O70 | O70:H- | 1303 | FN995094 | Leimbach *et al*. 2010, unpublished |
| 70 | O71 | O71 | - | GU445927 | [24] |
| 71 | O73 | O73:K-:H31 | G1275 | DQ000313.1 | [25] |
| 72 | O74 | O74:K-:H39 | E3a | KJ778807 | This study |
| 73 | O75 | O75:K95:H5 | E3b | KJ778786 | This study |
| 74 | O76 | O76 | E5d | AB612031 | [7] |
| 75 | O77 | O77:K96:H- | E1020-72 | DQ000314.1 | [25] |
| 76 | O78 | O78:H- | E38 | KJ778787 | This study |
| 77 | O79 | O79:K-:H40 | E49 | KJ778790 | This study |
| 78 | O80 | O80:K-:H26 | E71 | AB812032 | [7] |
| 79 | O81 | O81:K97:H- | H5 | KJ778811 | This study |
| 80 | O82 | O82:K-:H- | H14 | AB812034 | [7] |
| 81 | O83 | O83:K-:H31 | H17a | KJ778808 | This study |
| 82 | O84 | O84:K-:H21 | H19 | KJ778809 | This study |
| 83 | O85 | O85:K-:H1 | H23 | KJ778791 | This study |
| 84 | O86 | O86:H? | G1275 | AY670704 | [26] |
| 85 | O87 | O87 | - | EU294177 | [10] |
| 86 | O88 | O88:K-:H25 | H53 | KJ778812 | This study |
| 87 | O89 | O89:K-:H16 | H68 | KJ755555 | This study |
| 88 | O90 | O90:K-:H- | H77 | AB812039 | [7] |
| 89 | O91 | O91 | ECA95 | AY035396 | [27] |
| 90 | O92 | O92:K--:H33 | H308a | AB812040 | [7] |
| 91 | O93 | O93 | 2885-1 | AB812041 | [7] |
| 92 | O95 | O95:K+:H33 | H311a | KJ755556 | This study |
| 93 | O96 | O96:K-:H19 | H319 | KJ778788 | This study |
| 94 | O97 | O97:K-:H- | H320a | KJ778810 | This study |
| 95 | O98 | O98: K?:H8 | - | DQ180602 | [28] |
| 96 | O99 | O99 | G1251 | FJ940773 | [29] |
| 97 | O100 | O100:K-:H2 | H509a | KJ778805 | This study |
| 98 | O101 | O101:K-:H33 | H510a | KJ778806 | This study |
| 99 | O102 | O102 | G3100 | JX087966 | [30] |
| 100 | O103 | O103:K+:H8 | H515b | AY532664 | [31] |
| 101 | O104 | O104:K-:H12 | H519 | AF361371 | [32] |
| 102 | O105 | O105 | - | EU294171 | [10] |
| 103 | O106 | O106:K-:H33 | H521a | DQ000315.1 | [25] |
| 104 | O107 | O107:K98:H27 | H705 | EU694095 | [33] |
| 105 | O108 | O108:K-:H10 | H708b | KP710597 | This study |
| 106 | O109 | O109:K-:H19 | H709c | HM485572 | [34] |
| 107 | O110 | O110:K-:H39 | H711c | AB812049 | [7] |
| 108 | O111 | O111:H- | Stoke W; M92 | AF078736 | [35] |
| 109 | O112ab | O112ab:H18 | 1411-50 | EU296413 | [10] |
| 110 | O112ac | O112ac:H- | Guanabara (M194) | EU296405 | [10] |
| 111 | O113 | O113:H21 | 98NK2 | AF172324 | [36] |
| 112 | O114 | O114:H32 | G1088 | AY573377 | [37] |
| 113 | O115 | O115:K-:H18 | 27w | GU068041 | [38] |
| 114 | O116 | O116:K+:H10 | 28w | AB812051 | [7] |
| 115 | O117 | O117:K98:H4 | 30w | EU694096 | [33] |
| 116 | O118 | O118:K-:H- | 31w | DQ990684 | [39] |
| 117 | O119 | O119 | - | GQ499368 | [15] |
| 118 | O120 | O120:K18a:H6 | 35w | AB812052 | [7] |
| 119 | O121 | O121:K-:H10 | 39w | AY208937 | [40] |
| 120 | O123 | O123:K-:H16 | 43w | DQ676934 | [41] |
| 121 | O124 | O124 | - | EU296419 | [10] |
| 122 | O125ab | O125ab:H19 | 2745-53 | KP835694 | This study |
| 123 | O125ac | O125ac:H6 | 2129-54 | KP835695 | This study |
| 124 | O126 | O126:H2 | E611 | DQ465248 | [42] |
| 125 | O127 | O127:K63(B8) | G1094 | AY493508 | [43] |
| 126 | O128 | O128:B12 | - | AY217096 | [44] |
| 127 | O129 | O129 | - | EU296424 | [10] |
| 128 | O130 | O130 | - | EU296421 | [10] |
| 129 | O131 | O131:K-:H26 | S239 | KJ755544 | This study |
| 130 | O132 | O132:K+:H28 | N87 | KJ755553 | This study |
| 131 | O133 | O133:K-:H29 | N282 | KJ710509 | This study |
| 132 | O134 | O134:K-:H35 | 4370-53 | KJ755545 | This study |
| 133 | O135 | O135 | - | EU296423 | [10] |
| 134 | O136 | O136:H- | 1111-55 | KJ755546 | This study |
| 135 | O137 | O137:H41 | RVC1787 | KJ755547 | This study |
| 136 | O138 | O138:H- | CDC62-57 | DQ109551 | [45] |
| 137 | O139 | O139:K12:H1 | CDC63-57 | KJ755548 | This study |
| 138 | O140 | O140:K-:H43 | CDC149-51 | KJ755552 | This study |
| 139 | O141 | O141ac:H4 | RVC2907 | DQ868765 | [46] |
| 140 | O142 | O142:H6 | C771 | KJ755549 | This study |
| 141 | O143 | O143 | - | EU294164 | [10] |
| 142 | O144 | O144:K-:H- | 1624-56 | KJ755550 | This study |
| 143 | O145 | O145 | G1100 | AY647260 | [47] |
| 144 | O146 | O146:K-:H21 | CDC2950-54 | DQ465249 | [42] |
| 145 | O147 | O147 | - | DQ868766 | [46] |
| 146 | O148 | O148:H28 | E519-66 | DQ167407 | [48] |
| 147 | O149 | O149:H10 | - | DQ091854 | [49] |
| 148 | O150 | O150 | - | EU294168 | [10] |
| 149 | O151 | O151:K-:H10 | 880-67 | DQ990685 | [39] |
| 150 | O152 | O152 | - | EU294170 | [10] |
| 151 | O153 | O153:K-:H7 | 14097 | KJ755551 | This study |
| 152 | O154 | O154:K94:H4 | E1541-68 | AB812064 | [7] |
| 153 | O155 | O155:H9 | G1106 | AY657020 | [20] |
| 154 | O156 | O156:K-:H47 | E1585-68 | KJ755559 | This study |
| 155 | O157 | O157:H7 | C664-1992 | AF061251 | [50] |
| 156 | O158 | O158:K-:H23 | E1020-72 | GU068044 | [38] |
| 157 | O159 | O159 | - | EU294176 | [10] |
| 158 | O160 | O160:K-:H34 | E110-69 | KJ755560 | This study |
| 159 | O161 | O161 | G1254 | GU220361 | [21] |
| 160 | O162 | O162:K-:H10 | 10B1-1 | AB812067 | [7] |
| 161 | O163 | O163:K-:H19 | SN3B-1 | KP710593 | This study |
| 162 | O164 | O164 | - | EU296420 | [10] |
| 163 | O165 | O165:K-:H- | E78634 | GU068045 | [38] |
| 164 | O166 | O166 | G1216 | GU299794 | [51] |
| 165 | O167 | O167 | - | EU296408 | [10] |
| 166 | O168 | O168 | - | EU296403 | [10] |
| 167 | O169 | O169:K-:H8 | 1792-54 | KJ778796 | This study |
| 168 | O170 | O170:K-:H1 | 745-54 | KJ778797 | This study |
| 169 | O171 | O171:K-:H2 | 198 | KJ739598 | This study |
| 170 | O172 | O172 | G1092 | AY545992 | [52] |
| 171 | O173 | O173: K-:H- | L119B-10 | GU068046 | [38] |
| 172 | O174 | O174:K-:H27 | 2531-54 | DQ008592 | [53] |
| 173 | O175 | O175:K-:H28 | 2533-54 | KJ739597 | This study |
| 174 | O176 | OX176:H- | E29518-83 | KJ778798 | This study |
| 175 | O177 | OX177:H25 | E40874-85 | DQ008593 | [53] |
| 176 | O178 | OX178:H7 | E54071-88 | KJ778799 | This study |
| 177 | O179 | OX179:H8 | E43478 | KJ778800 | This study |
| 178 | O180 | OX180:H- | 86-381 | JQ751058 | [54] |
| 179 | O181 | O181:H49 | 92-1250 | KJ778801 | This study |
| 180 | O182 | O182:K48:H25 | SSI 81930 | KJ778802 | This study |
| 181 | O183 | O183:H18 | 99-2442 | AB627352.1 | [55] |
| 182 | O184 | O184:K-:H11 | 99-4473 | AB812080.1 | [7] |
| 183 | O185 | O185:H28 | 99-6301 | AB812081 | [7] |
| 184 | O186 | O186:K-:H- | SSI 81934 | KP710595 | This study |
| 185 | O187 | O187:K-:H52 | SSI 81829 | KJ739600 | This study |
| 186 | OX6 | OX6:H4 | 244-54 | KJ778794 | This study |
| 187 | OX9 | OX9:H10 | 3461-54 | KJ778795 | This study |
| 188 | OX10 | OX10:H34 | 6941-60 | KJ778792 | This study |
| 189 | OX13 | OX13:H10 | 22-56 | KP710591 | This study |
| 190 | OX18 | OX18:H12 | PF1912 | KJ710507 | This study |
| 191 | OX19 | OX19:H21 | PDL-39A | KP868751 | This study |
| 192 | OX21 | OX21:H14 | Pur599 | KJ739596 | This study |
| 193 | OX25 | OX25:H6 | F6432 | KP710594 | This study |
| 194 | OX28 | OX28:H27 | 7026N | KT207929 | This study |
| 195 | OX38 | OX38:H2 | PF11-6E | KJ739599 | This study |
| 196 | OX43 | OX43:H19 | 8547 | KP835691 | This study |

**References**

1. Li D, Liu B, Chen M, Guo D, Guo X, Liu F, et al. A multiplex PCR method to detect 14 Escherichia coli serogroups associated with urinary tract infections. Journal of microbiological methods. 2010;82(1):71-7. doi: 10.1016/j.mimet.2010.04.008. PubMed PMID: 20434495.

2. Fratamico PM, Yan X, Liu Y, DebRoy C, Byrne B, Monaghan A, et al. Escherichia coli serogroup O2 and O28ac O-antigen gene cluster sequences and detection of pathogenic E. coli O2 and O28ac by PCR. Canadian journal of microbiology. 2010;56(4):308-16. doi: 10.1139/w10-010. PubMed PMID: 20453897.

3. Ren Y, Liu B, Cheng J, Liu F, Feng L, Wang L. Characterization of Escherichia coli O3 and O21 O antigen gene clusters and development of serogroup-specific PCR assays. Journal of microbiological methods. 2008;75(2):329-34. doi: 10.1016/j.mimet.2008.07.010. PubMed PMID: 18700154.

4. D'Souza JM, Samuel GN, Reeves PR. Evolutionary origins and sequence of the Escherichia coli O4 O-antigen gene cluster. FEMS microbiology letters. 2005;244(1):27-32. doi: 10.1016/j.femsle.2005.01.012. PubMed PMID: 15727817.

5. Grozdanov L, Zahringer U, Blum-Oehler G, Brade L, Henne A, Knirel YA, et al. A single nucleotide exchange in the wzy gene is responsible for the semirough O6 lipopolysaccharide phenotype and serum sensitivity of Escherichia coli strain Nissle 1917. Journal of bacteriology. 2002;184(21):5912-25. PubMed PMID: 12374825; PubMed Central PMCID: PMC135379.

6. Marolda CL, Feldman MF, Valvano MA. Genetic organization of the O7-specific lipopolysaccharide biosynthesis cluster of Escherichia coli VW187 (O7:K1). Microbiology. 1999;145 ( Pt 9):2485-95. PubMed PMID: 10517601.

7. Iguchi A, Iyoda S, Kikuchi T, Ogura Y, Katsura K, Ohnishi M, et al. A complete view of the genetic diversity of the Escherichia coli O-antigen biosynthesis gene cluster. DNA Res. 2015;22(1):101-7. doi: 10.1093/dnares/dsu043. PubMed PMID: 25428893; PubMed Central PMCID: PMC4379981.

8. Kido N, Torgov VI, Sugiyama T, Uchiya K, Sugihara H, Komatsu T, et al. Expression of the O9 polysaccharide of Escherichia coli: sequencing of the E. coli O9 rfb gene cluster, characterization of mannosyl transferases, and evidence for an ATP-binding cassette transport system. Journal of bacteriology. 1995;177(8):2178-87. PubMed PMID: 7536735; PubMed Central PMCID: PMC176863.

9. Li Y, Perepelov AV, Guo D, Shevelev SD, Senchenkova SN, Shahskov AS, et al. Structural and genetic relationships of two pairs of closely related O-antigens of Escherichia coli and Salmonella enterica: E. coli O11/S. enterica O16 and E. coli O21/S. enterica O38. FEMS immunology and medical microbiology. 2011;61(3):258-68. doi: 10.1111/j.1574-695X.2010.00771.x. PubMed PMID: 21205000.

10. Liu B, Knirel YA, Feng L, Perepelov AV, Senchenkova SN, Wang Q, et al. Structure and genetics of Shigella O antigens. FEMS microbiology reviews. 2008;32(4):627-53. doi: 10.1111/j.1574-6976.2008.00114.x. PubMed PMID: 18422615.

11. Beutin L, Tao J, Feng L, Krause G, Zimmermann S, Gleier K, et al. Sequence analysis of the Escherichia coli O15 antigen gene cluster and development of a PCR assay for rapid detection of intestinal and extraintestinal pathogenic E. coli O15 strains. Journal of clinical microbiology. 2005;43(2):703-10. doi: 10.1128/JCM.43.2.703-710.2005. PubMed PMID: 15695667; PubMed Central PMCID: PMC548065.

12. Fratamico P, DebRoy C, Liu Y. The DNA Sequence of the Escherichia coli O22 O-Antigen Gene Cluster and Detection of Pathogenic Strains Belonging to E. coli Serogroups O22 and O91 by Multiplex PCR Assays Targeting Virulence Genes and Genes in the Respective O-Antigen Gene Clusters. Food Anal Methods. 2009;2(3):169-79. doi: 10.1007/s12161-008-9046-z.

13. Wang Q, Wang S, Beutin L, Cao B, Feng L, Wang L. Development of a DNA microarray for detection and serotyping of enterotoxigenic Escherichia coli. Journal of clinical microbiology. 2010;48(6):2066-74. doi: 10.1128/JCM.02014-09. PubMed PMID: 20351209; PubMed Central PMCID: PMCPMC2884529.

14. D'Souza JM, Wang L, Reeves P. Sequence of the Escherichia coli O26 O antigen gene cluster and identification of O26 specific genes. Gene. 2002;297(1-2):123-7. PubMed PMID: 12384293.

15. Liu B, Wu F, Li D, Beutin L, Chen M, Cao B, et al. Development of a serogroup-specific DNA microarray for identification of Escherichia coli strains associated with bovine septicemia and diarrhea. Veterinary microbiology. 2010;142(3-4):373-8. doi: 10.1016/j.vetmic.2009.10.019. PubMed PMID: 19932572.

16. DebRoy C, Fratamico PM, Roberts E, Davis MA, Liu Y. Development of PCR assays targeting genes in O-antigen gene clusters for detection and identification of Escherichia coli O45 and O55 serogroups. Applied and environmental microbiology. 2005;71(8):4919-24. doi: 10.1128/AEM.71.8.4919-4924.2005. PubMed PMID: 16085897; PubMed Central PMCID: PMC1183307.

17. Feng L, Senchenkova SN, Yang J, Shashkov AS, Tao J, Guo H, et al. Synthesis of the heteropolysaccharide O antigen of Escherichia coli O52 requires an ABC transporter: structural and genetic evidence. Journal of bacteriology. 2004;186(14):4510-9. doi: 10.1128/JB.186.14.4510-4519.2004. PubMed PMID: 15231783; PubMed Central PMCID: PMC438562.

18. Wang L, Huskic S, Cisterne A, Rothemund D, Reeves PR. The O-antigen gene cluster of Escherichia coli O55:H7 and identification of a new UDP-GlcNAc C4 epimerase gene. Journal of bacteriology. 2002;184(10):2620-5. PubMed PMID: 11976290; PubMed Central PMCID: PMC135022.

19. Cheng J, Wang Q, Wang W, Wang Y, Wang L, Feng L. Characterization of E. coli O24 and O56 O antigen gene clusters reveals a complex evolutionary history of the O24 gene cluster. Current microbiology. 2006;53(6):470-6. doi: 10.1007/s00284-006-0032-7. PubMed PMID: 17072668.

20. Guo H, Kong Q, Cheng J, Wang L, Feng L. Characterization of the Escherichia coli O59 and O155 O-antigen gene clusters: the atypical wzx genes are evolutionary related. FEMS microbiology letters. 2005;248(2):153-61. doi: 10.1016/j.femsle.2005.05.036. PubMed PMID: 15990253.

21. Li X, Perepelov AV, Wang Q, Senchenkova SN, Liu B, Shevelev SD, et al. Structural and genetic characterization of the O-antigen of Escherichia coli O161 containing a derivative of a higher acidic diamino sugar, legionaminic acid. Carbohydrate research. 2010;345(11):1581-7. doi: 10.1016/j.carres.2010.04.008. PubMed PMID: 20510395.

22. Liu Y, Yan X, DebRoy C, Fratamico PM, Needleman DS, Li RW, et al. Escherichia coli O-Antigen Gene Clusters of Serogroups O62, O68, O131, O140, O142, and O163: DNA Sequences and Similarity between O62 and O68, and PCR-Based Serogrouping. Biosensors (Basel). 2015;5(1):51-68. doi: 10.3390/bios5010051. PubMed PMID: 25664526; PubMed Central PMCID: PMC4384082.

23. Cheng J, Liu B, Bastin DA, Han W, Wang L, Feng L. Genetic characterization of the Escherichia coli O66 antigen and functional identification of its wzy gene. J Microbiol. 2007;45(1):69-74. PubMed PMID: 17342059.

24. Hu B, Perepelov AV, Liu B, Shevelev SD, Guo D, Senchenkova SN, et al. Structural and genetic evidence for the close relationship between Escherichia coli O71 and Salmonella enterica O28 O-antigens. FEMS immunology and medical microbiology. 2010;59(2):161-9. doi: 10.1111/j.1574-695X.2010.00676.x. PubMed PMID: 20482625.

25. Wang W, Perepelov AV, Feng L, Shevelev SD, Wang Q, Senchenkova SN, et al. A group of Escherichia coli and Salmonella enterica O antigens sharing a common backbone structure. Microbiology. 2007;153(Pt 7):2159-67. doi: 10.1099/mic.0.2007/004192-0. PubMed PMID: 17600060.

26. Feng L, Han W, Wang Q, Bastin DA, Wang L. Characterization of Escherichia coli O86 O-antigen gene cluster and identification of O86-specific genes. Veterinary microbiology. 2005;106(3-4):241-8. doi: 10.1016/j.vetmic.2004.12.021. PubMed PMID: 15778030.

27. Perelle S, Dilasser F, Grout J, Fach P. Identification of the O-antigen biosynthesis genes of Escherichia coli O91 and development of a O91 PCR serotyping test. Journal of applied microbiology. 2002;93(5):758-64. PubMed PMID: 12392520.

28. Cunneen MM, Reeves PR. The Yersinia kristensenii O11 O-antigen gene cluster was acquired by lateral gene transfer and incorporated at a novel chromosomal locus. Mol Biol Evol. 2007;24(6):1355-65. doi: 10.1093/molbev/msm058. PubMed PMID: 17400574.

29. Perepelov AV, Li D, Liu B, Senchenkova SN, Guo D, Shevelev SD, et al. Structural and genetic characterization of Escherichia coli O99 antigen. FEMS immunology and medical microbiology. 2009;57(1):80-7. doi: 10.1111/j.1574-695X.2009.00584.x. PubMed PMID: 19682076.

30. Perepelov AV, Wang Q, Senchenkova SN, Gong Y, Shashkov AS, Wang L, et al. Structure and gene cluster of the O-antigen of Escherichia coli O102. Carbohydrate research. 2012;361:73-7. doi: 10.1016/j.carres.2012.07.024. PubMed PMID: 22982615.

31. Fratamico PM, DebRoy C, Strobaugh TP, Jr., Chen CY. DNA sequence of the Escherichia coli O103 O antigen gene cluster and detection of enterohemorrhagic E. coli O103 by PCR amplification of the wzx and wzy genes. Canadian journal of microbiology. 2005;51(6):515-22. doi: 10.1139/w05-049. PubMed PMID: 16121232.

32. Wang L, Briggs CE, Rothemund D, Fratamico P, Luchansky JB, Reeves PR. Sequence of the E. coli O104 antigen gene cluster and identification of O104 specific genes. Gene. 2001;270(1-2):231-6. PubMed PMID: 11404020.

33. Wang Q, Perepelov AV, Feng L, Knirel YA, Li Y, Wang L. Genetic and structural analyses of Escherichia coli O107 and O117 O-antigens. FEMS immunology and medical microbiology. 2009;55(1):47-54. doi: 10.1111/j.1574-695X.2008.00494.x. PubMed PMID: 19040662.

34. Perepelov AV, Ni Z, Wang Q, Shevelev SD, Senchenkova SN, Shahskov AS, et al. Structure and gene cluster of the O-antigen of Escherichia coli O109; chemical and genetic evidences of the presence of L-RhaN3N derivatives in the O-antigens of E. coli O109 and O119. FEMS immunology and medical microbiology. 2011;61(1):47-53. doi: 10.1111/j.1574-695X.2010.00745.x. PubMed PMID: 20964722.

35. Bastin DA, Reeves PR. Sequence and analysis of the O antigen gene (rfb) cluster of Escherichia coli O111. Gene. 1995;164(1):17-23. PubMed PMID: 7590310.

36. Paton AW, Paton JC. Molecular characterization of the locus encoding biosynthesis of the lipopolysaccharide O antigen of Escherichia coli serotype O113. Infection and immunity. 1999;67(11):5930-7. PubMed PMID: 10531250; PubMed Central PMCID: PMC96976.

37. Feng L, Wang W, Tao J, Guo H, Krause G, Beutin L, et al. Identification of Escherichia coli O114 O-antigen gene cluster and development of an O114 serogroup-specific PCR assay. Journal of clinical microbiology. 2004;42(8):3799-804. doi: 10.1128/JCM.42.8.3799-3804.2004. PubMed PMID: 15297533; PubMed Central PMCID: PMC497616.

38. Wang Q, Ruan X, Wei D, Hu Z, Wu L, Yu T, et al. Development of a serogroup-specific multiplex PCR assay to detect a set of Escherichia coli serogroups based on the identification of their O-antigen gene clusters. Molecular and cellular probes. 2010;24(5):286-90. doi: 10.1016/j.mcp.2010.06.002. PubMed PMID: 20561581.

39. Liu Y, Fratamico P, Debroy C, Bumbaugh AC, Allen JW. DNA sequencing and identification of serogroup-specific genes in the Escherichia coli O118 O antigen gene cluster and demonstration of antigenic diversity but only minor variation in DNA sequence of the O antigen clusters of E. coli O118 and O151. Foodborne pathogens and disease. 2008;5(4):449-57. doi: 10.1089/fpd.2008.0096. PubMed PMID: 18673069.

40. Fratamico PM, Briggs CE, Needle D, Chen CY, DebRoy C. Sequence of the Escherichia coli O121 O-antigen gene cluster and detection of enterohemorrhagic E. coli O121 by PCR amplification of the wzx and wzy genes. Journal of clinical microbiology. 2003;41(7):3379-83. PubMed PMID: 12843098; PubMed Central PMCID: PMC165269.

41. Beutin L, Wang Q, Naumann D, Han W, Krause G, Leomil L, et al. Relationship between O-antigen subtypes, bacterial surface structures and O-antigen gene clusters in Escherichia coli O123 strains carrying genes for Shiga toxins and intimin. Journal of medical microbiology. 2007;56(Pt 2):177-84. doi: 10.1099/jmm.0.46775-0. PubMed PMID: 17244797.

42. Liu Y, DebRoy C, Fratamico P. Sequencing and analysis of the Escherichia coli serogroup O117, O126, and O146 O-antigen gene clusters and development of PCR assays targeting serogroup O117-, O126-, and O146-specific DNA sequences. Molecular and cellular probes. 2007;21(4):295-302. doi: 10.1016/j.mcp.2007.03.002. PubMed PMID: 17452091.

43. Perepelov AV, Liu B, Senchenkova SN, Shevelev SD, Feng L, Shashkov AS, et al. The O-antigen of Salmonella enterica O13 and its relation to the O-antigen of Escherichia coli O127. Carbohydrate research. 2010;345(12):1808-11. doi: 10.1016/j.carres.2010.05.015. PubMed PMID: 20594547.

44. Shao J, Li M, Jia Q, Lu Y, Wang PG. Sequence of Escherichia coli O128 antigen biosynthesis cluster and functional identification of an alpha-1,2-fucosyltransferase. FEBS letters. 2003;553(1-2):99-103. PubMed PMID: 14550554.

45. Wang L, Liu B, Kong Q, Steinruck H, Krause G, Beutin L, et al. Molecular markers for detection of pathogenic Escherichia coli strains belonging to serogroups O 138 and O 139. Veterinary microbiology. 2005;111(3-4):181-90. doi: 10.1016/j.vetmic.2005.10.006. PubMed PMID: 16280204.

46. Han W, Liu B, Cao B, Beutin L, Kruger U, Liu H, et al. DNA microarray-based identification of serogroups and virulence gene patterns of Escherichia coli isolates associated with porcine postweaning diarrhea and edema disease. Applied and environmental microbiology. 2007;73(12):4082-8. doi: 10.1128/AEM.01820-06. PubMed PMID: 17449692; PubMed Central PMCID: PMC1932722.

47. Feng L, Senchenkova SN, Tao J, Shashkov AS, Liu B, Shevelev SD, et al. Structural and genetic characterization of enterohemorrhagic Escherichia coli O145 O antigen and development of an O145 serogroup-specific PCR assay. Journal of bacteriology. 2005;187(2):758-64. doi: 10.1128/JB.187.2.758-764.2005. PubMed PMID: 15629947; PubMed Central PMCID: PMC543545.

48. Feng L, Perepelov AV, Zhao G, Shevelev SD, Wang Q, Senchenkova SN, et al. Structural and genetic evidence that the Escherichia coli O148 O antigen is the precursor of the Shigella dysenteriae type 1 O antigen and identification of a glucosyltransferase gene. Microbiology. 2007;153(Pt 1):139-47. doi: 10.1099/mic.0.2006/001107-0. PubMed PMID: 17185542.

49. Goswami P, Gyles C, Friendship R, Poppe C, Vinogradov E, Boerlin P. The Escherichia coli O149 rfb gene cluster and its use for the detection of porcine E. coli O149 by real-time PCR. Veterinary microbiology. 2010;141(1-2):120-6. doi: 10.1016/j.vetmic.2009.08.017. PubMed PMID: 19733452.

50. Wang L, Reeves PR. Organization of Escherichia coli O157 O antigen gene cluster and identification of its specific genes. Infection and immunity. 1998;66(8):3545-51. PubMed PMID: 9673232; PubMed Central PMCID: PMC108385.

51. Liu B, Perepelov AV, Li D, Senchenkova SN, Han Y, Shashkov AS, et al. Structure of the O-antigen of Salmonella O66 and the genetic basis for similarity and differences between the closely related O-antigens of Escherichia coli O166 and Salmonella O66. Microbiology. 2010;156(Pt 6):1642-9. doi: 10.1099/mic.0.037325-0. PubMed PMID: 20185508.

52. Guo H, Feng L, Tao J, Zhang C, Wang L. Identification of Escherichia coli O172 O-antigen gene cluster and development of a serogroup-specific PCR assay. Journal of applied microbiology. 2004;97(1):181-90. doi: 10.1111/j.1365-2672.2004.02305.x. PubMed PMID: 15186455.

53. Beutin L, Kong Q, Feng L, Wang Q, Krause G, Leomil L, et al. Development of PCR assays targeting the genes involved in synthesis and assembly of the new Escherichia coli O 174 and O 177 O antigens. Journal of clinical microbiology. 2005;43(10):5143-9. doi: 10.1128/JCM.43.10.5143-5149.2005. PubMed PMID: 16207976; PubMed Central PMCID: PMC1248525.

54. Wang Q, Perepelov AV, Beutin L, Senchenkova SN, Xu Y, Shashkov AS, et al. Structural and genetic characterization of the Escherichia coli O180 O antigen and identification of a UDP-GlcNAc 6-dehydrogenase. Glycobiology. 2012;22(10):1321-31. doi: 10.1093/glycob/cws098. PubMed PMID: 22730467.

55. Iguchi A, Iyoda S, Seto K, Ohnishi M, Group ES. Emergence of a novel Shiga toxin-producing Escherichia coli O serogroup cross-reacting with Shigella boydii type 10. Journal of clinical microbiology. 2011;49(10):3678-80. doi: 10.1128/JCM.01197-11. PubMed PMID: 21865424; PubMed Central PMCID: PMC3187333.
