# Supplementary material for: Correction: Comparison of O-Antigen Gene Clusters of All O-Serogroups of Escherichia coli and Proposal for Adopting a New Nomenclature for O-Typing
Source: PLoS One. 2016 Apr 27;11(4):e0154551. doi: 10.1371/journal.pone.0154551 (PMC4847782; doi:10.1371/journal.pone.0154551)
Supplement: S1 Fig — The O-AGCs of all 196 O- and OX-groups are diagrammatically represented. The nucleotide sequences of 71 O-groups marked with asterisk and in bold font were determined in the present investigation. (PDF) [file pone.0154551.s001.pdf]

PLOS ONE

Comparison of O-Antigen Gene Clusters of all O-Serogroups of Escherichia coli and Proposal for adopting a new nomenclature for O-Typing of Escherichia coli.

--Manuscript Draft--

**Manuscript Number:**	PONE-D-16-07782
**Article Type:**	Author Correction
**Corresponding Author:**	Chitrita DebRoy<br>The Pennsylvania State University<br>University Park, Pennsylvania UNITED STATES
**First Author:**	Chitrita DebRoy
**Order of Authors:**	Chitrita DebRoy

Powered by Editorial Manager® and ProduXion Manager® from Aries Systems Corporation

Correction

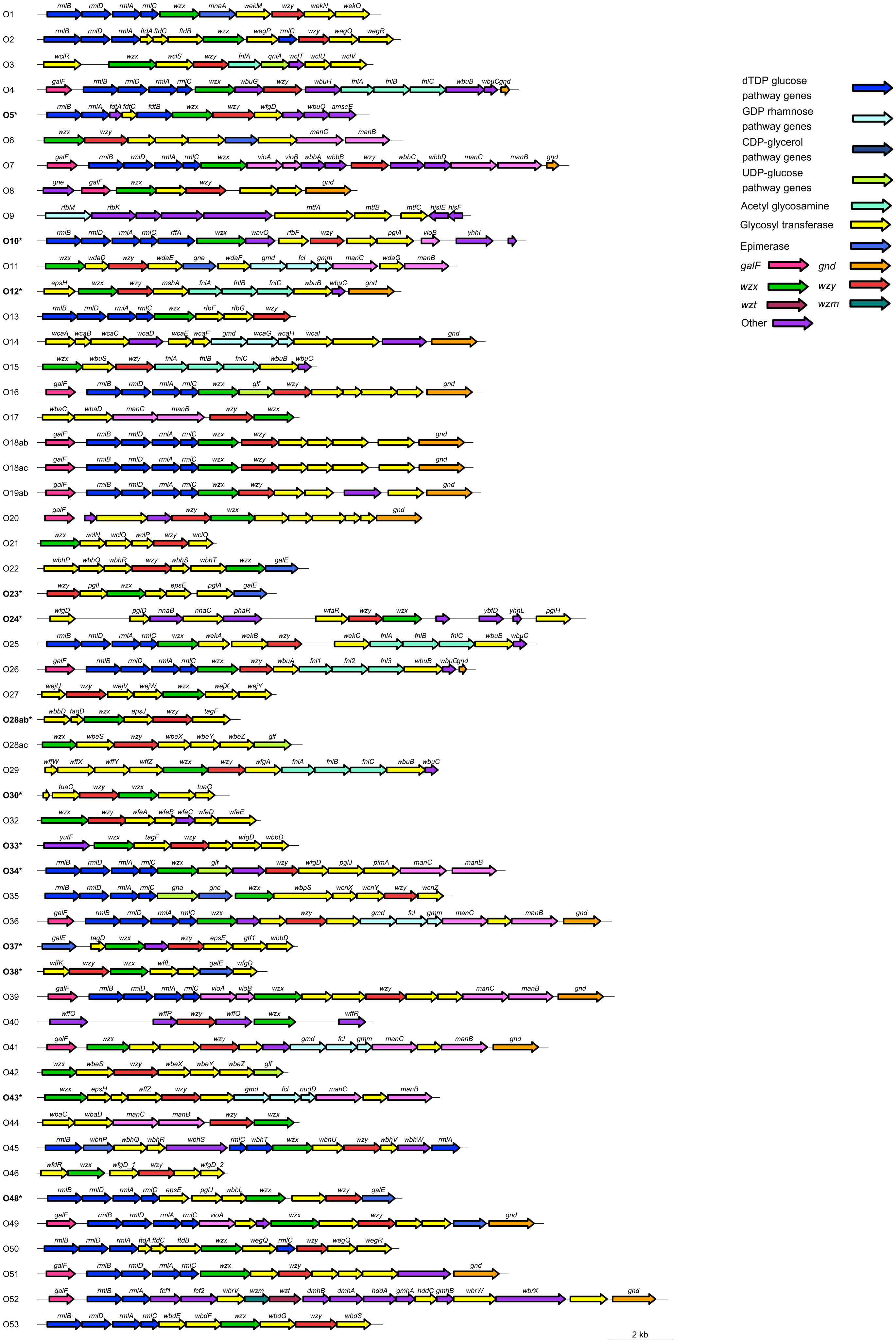

O1 rmlB rmlD rmlA rmlC wzx mnaA wekM wzy wekN wekO
O2 rmlB rmlD rmlA ftdA ftdC ftdB wzx wegP rmlC wzy wegQ wegR
O3 wclR wzx wclS wzy fnlA qnlA wclT wclU wclV
O4 galF rmlB rmlD rmlA rmlC wzx wbuG wzy wbuH fnlA fnlB fnlC wbuB wbuC gnd
O5* rmlB rmlA fdtA fdtC fdtB wzx wzy wfgD wbuQ amsE
O6 wzx wzy manC manB
O7 galF rmlB rmlD rmlA rmlC wzx vioA vioB wbbA wbbB wzy wbbC wbbD manC manB gnd
O8 gne galF wzx wzy gnd
O9 rfbM rfbK mtfA mtfB mtfC hisIE hisF
O10* rmlB rmlD rmlA rmlC rffA wzx wavQ rfbF wzy pglA vioB yhhI
O11 wzx wdaD wzy wdaE gne wdaF gmd fcl gmm manC wdaG manB
O12* epsH wzx wzy mshA fnlA fnlB fnlC wbuB wbuC gnd
O13 rmlB rmlD rmlA rmlC wzx rfbF rfbG wzy
O14 wcaA wcaB wcaC wcaD wcaE wcaF gmd wcaG wcaH wcaI gnd
O15 wzx wbuS wzy fnlA fnlB fnlC wbuB wbuC
O16 galF rmlB rmlD rmlA rmlC wzx glf wzy gnd
O17 wbaC wbaD manC manB wzy wzx
O18ab galF rmlB rmlD rmlA rmlC wzx wzy gnd
O18ac galF rmlB rmlD rmlA rmlC wzx wzy gnd
O19ab galF rmlB rmlD rmlA rmlC wzx wzy gnd
O20 galF wzy wzx gnd
O21 wzx wclN wclO wclP wzy wclQ
O22 wbhP wbhQ wbhR wzy wbhS wbhT wzx galE
O23* wzy pglI wzx epsE pglA galE
O24* wfgD pglD nnaB nnaC phaR wfaR wzy wzx ybfD yhhL pglH
O25 rmlB rmlD rmlA rmlC wzx wekA wekB wzy wekC fnlA fnlB fnlC wbuB wbuC
O26 galF rmlB rmlD rmlA rmlC wzx wzy wbuA fnl1 fnl2 fnl3 wbuB wbuC gnd
O27 wejU wzy wejV wejW wzx wejX wejY
O28ab* wbbD tagD wzx epsJ wzy tagF
O28ac wzx wbeS wzy wbeX wbeY wbeZ glf
O29 wffW wffX wffY wffZ wzx wzy wfgA fnlA fnlB fnlC wbuB wbuC
O30* tuaC wzy wzx tuaG
O32 wzx wzy wfeA wfeB wfeC wfeD wfeE
O33* yutF wzx tagF wzy wfgD wbbD
O34* rmlB rmlD rmlA rmlC wzx glf wzy wfgD pglJ pimA manC manB
O35 rmlB rmlD rmlA rmlC gna gne wzx wbpS wcnX wcnY wzy wcnZ
O36 galF rmlB rmlD rmlA rmlC wzx wzy gmd fcl gmm manC manB gnd
O37* galE tagD wzx wzy epsE gtf1 wbbD
O38* wffK wzy wzx wffL galE wfgD
O39 galF rmlB rmlD rmlA rmlC vioA vioB wzx wzy manC manB gnd
O40 wffO wffP wzy wffQ wzx wffR
O41 galF wzx wzy gmd fcl gmm manC manB gnd
O42 wzx wbeS wzy wbeX wbeY wbeZ glf
O43* wzx epsH wffZ wzy gmd fcl nudD manC manB
O44 wbaC wbaD manC manB wzy wzx
O45 rmlB wbhP wbhQ wbhR wbhS rmlC wbhT wzx wbhU wzy wbhV wbhW rmlA
O46 wfdR wzx wfgD_1 wzy wfgD_2
O48* rmlB rmlD rmlA rmlC epsE pglJ wbbL wzx wzy galE
O49 galF rmlB rmlD rmlA rmlC vioA wzx wzy gnd
O50 rmlB rmlD rmlA ftdA ftdC ftdB wzx wegQ rmlC wzy wegQ wegR
O51 galF rmlB rmlD rmlA rmlC wzx wzy gnd
O52 galF rmlB rmlA fcf1 fcf2 wbrV wzm wzt dmhB dmhA hddA gmhA hddC gmhB wbrW wbrX gnd
O53 rmlB rmlD rmlA rmlC wbdE wbdF wzx wbdG wzy wbdS
dTDP glucose pathway genes
GDP rhamnose pathway genes
CDP-glycerol pathway genes
UDP-glucose pathway genes
Acetyl glycosamine
Glycosyl transferase
Epimerase
galF
gnd
wzx
wzy
wzt
wzm
Other
2 kb

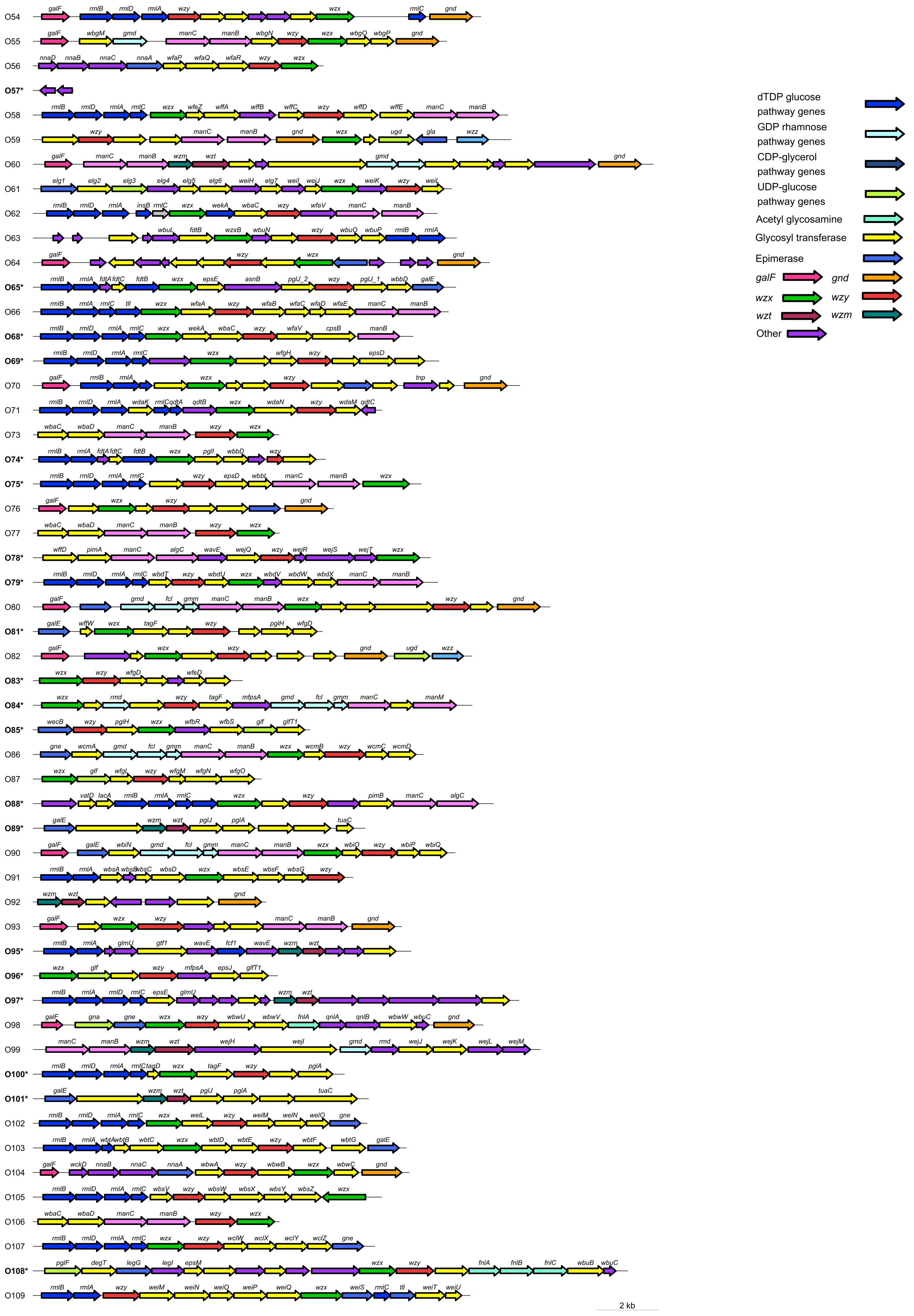
O54 galF rmlB rmlD rmlA wzy wzx rmlC gnd
O55 galF wbgM gmd manC manB wbgN wzy wzx wbgO wbgP gnd
O56 nnaD nnaB nnaC nnaA wfaP wfaQ wfaR wzy wzx
O57*
O58 rmlB rmlD rmlA rmlC wzx wfeZ wffA wffB wffC wzy wffD wffE manC manB
O59 wzy manC manB gnd wzx ugd gla wzz
O60 galF manC manB wzm wzt gmd gnd
O61 elg1 elg2 elg3 elg4 elg5 elg6 weiH elg7 weiI weiJ wzx weiK wzy weiL
O62 rmlB rmlD rmlA insB rmlC wzx wekA wbaC wzy wfaV manC manB
O63 wbuL fdtB wzxB wbuN wzy wbuO wbuP rmlB rmlA
O64 galF wzy wzx gnd
O65* rmlB rmlA fdtA fdtC fdtB wzx epsE asnB pglJ_2 wzy pglJ_1 wbbD galE
O66 rmlB rmlA rmlC tll wzx wfaA wzy wfaB wfaC wfaD wfaE manC manB
O68* rmlB rmlD rmlA rmlC wzx wekA wbaC wzy wfaV cpsB manB
O69* rmlB rmlD rmlA rmlC wzx wfgH wzy epsD
O70 galF rmlB rmlA wzx wzy tnp gnd
O71 rmlB rmlD rmlA wdaK rmlC qdtA qdtB wzx wdaN wzy wdaM qdtC
O73 wbaC wbaD manC manB wzy wzx
O74* rmlB rmlA fdtA fdtC fdtB wzx pglI wbbD wzy
O75* rmlB rmlD rmlA rmlC wzy epsD wbbL manC manB wzx
O76 galF wzx wzy gnd
O77 wbaC wbaD manC manB wzy wzx
O78* wffD pimA manC algC wavE wejQ wzy wejR wejS wejT wzx
O79* rmlB rmlD rmlA rmlC wbdT wzy wbdU wzx wbdV wbdW wbdX manC manB
O80 galF gmd fcl gmm manC manB wzx wzy gnd
O81* galE wffW wzx tagF wzy pglH wfgD
O82 galF wzx wzy gnd ugd wzz
O83* wzx wzy wfgD wfeD
O84* wzx rmd wzy tagF mfpsA gmd fcl gmm manC manM
O85* wecB wzy pglH wzx wfbR wfbS glf glfT1
O86 gne wcmA gmd fcl gmm manC manB wzx wcmB wzy wcmC wcmD
O87 wzx glf wfgL wzy wfgM wfgN wfgO
O88* vatD lacA rmlB rmlA rmlC wzx wzy pimB manC algC
O89* galE wzm wzt pglJ pglA tuaC
O90 galF galE wbiN gmd fcl gmm manC manB wzx wbiO wzy wbiP wbiQ
O91 rmlB rmlA wbsA wbsB wbsC wbsD wzx wbsE wbsF wbsG wzy
O92 wzm wzt gnd
O93 galF wzx wzy manC manB gnd
O95* rmlB rmlA glmU gtf1 wavE fcf1 wavE wzm wzt
O96* wzx glf wzy mfpsA epsJ glfT1
O97* rmlB rmlA rmlD rmlC epsE glmU wzm wzt
O98 galF gna gne wzx wzy wbwU wbwV fnlA qnlA qnlB wbwW wbuC gnd
O99 manC manB wzm wzt wejH wejI gmd rmd wejJ wejK wejL wejM
O100* rmlB rmlD rmlA rmlC tagD wzx tagF wzy pglA
O101* galE wzm wzt pglJ pglA tuaC
O102 rmlB rmlD rmlA rmlC wzx weiL wzy weiM weiN weiO gne
O103 rmlB rmlA wbtA wbtB wbtC wzx wbtD wbtE wzy wbtF wbtG galE
O104 galF wckD nnaB nnaC nnaA wbwA wzy wbwB wzx wbwC gnd
O105 rmlB rmlD rmlA rmlC wbsV wzy wbsW wbsX wbsY wbsZ wzx
O106 wbaC wbaD manC manB wzy wzx
O107 rmlB rmlD rmlA rmlC wzx wzy wclW wclX wclY wclZ gne
O108* pglF degT legG legI epsM wzx wzy fnlA fnlB fnlC wbuB wbuC
O109 rmlB rmlA wzy weiM weiN weiO weiP weiQ wzx weiS rmlC tll weiT weiU
2 kb
dTDP glucose pathway genes
GDP rhamnose pathway genes
CDP-glycerol pathway genes
UDP-glucose pathway genes
Acetyl glycosamine
Glycosyl transferase
Epimerase
galF
gnd
wzx
wzy
wzt
wzm
Other

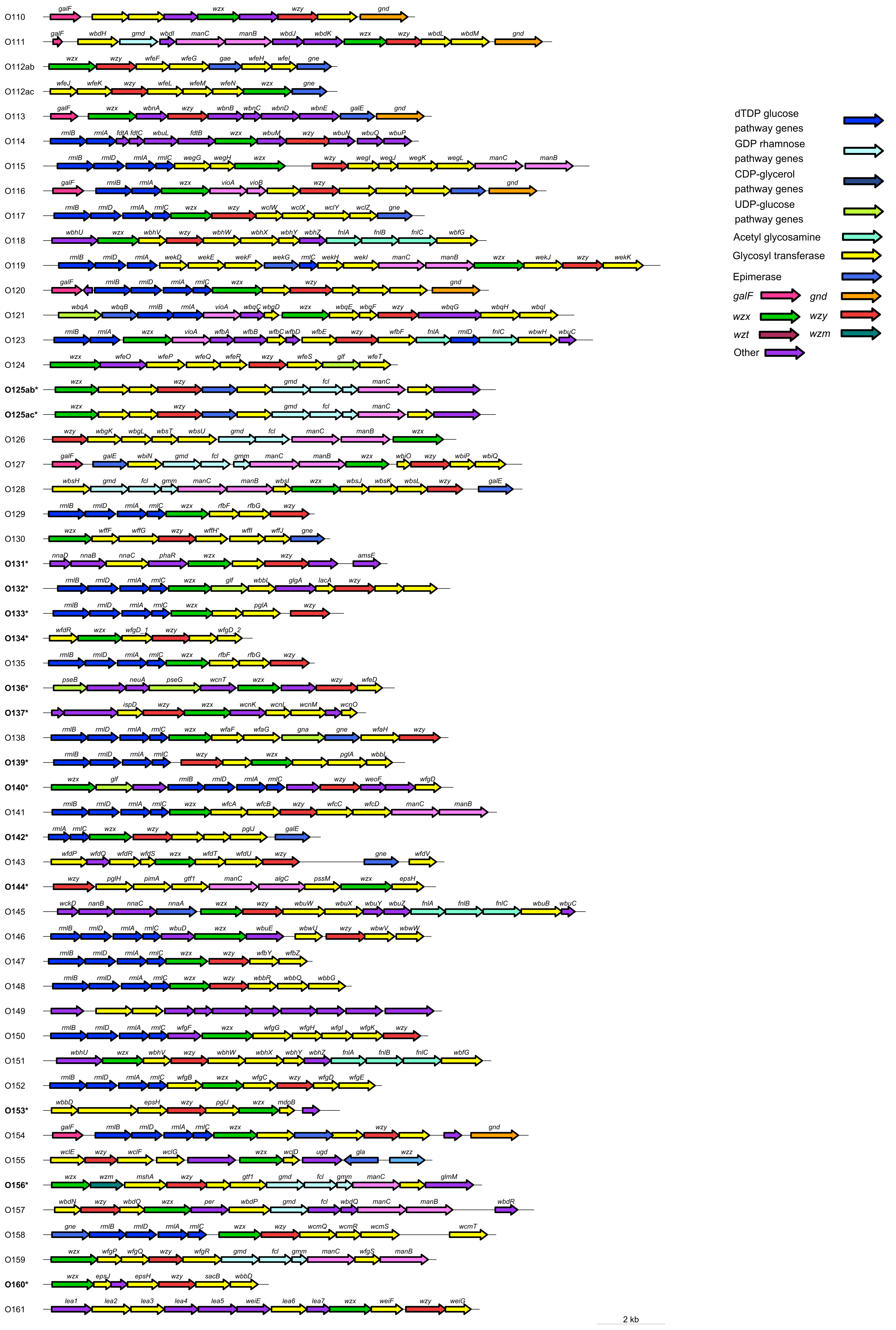
O110 galF wzx wzy gnd
O111 galF wbdH gmd wbdI manC manB wbdJ wbdK wzx wzy wbdL wbdM gnd
O112ab wzx wzy wfeF wfeG gae wfeH wfeI gne
O112ac wfeJ wfeK wzy wfeL wfeM wfeN wzx gne
O113 galF wzx wbnA wzy wbnB wbnC wbnD wbnE galE gnd
O114 rmlB rmlA fdtA fdtC wbuL fdtB wzx wbuM wzy wbuN wbuO wbuP
O115 rmlB rmlD rmlA rmlC wegG wegH wzx wzy wegI wegJ wegK wegL manC manB
O116 galF rmlB rmlA wzx vioA vioB wzy gnd
O117 rmlB rmlD rmlA rmlC wzx wzy wclW wclX wclY wclZ gne
O118 wbhU wzx wbhV wzy wbhW wbhX wbhY wbhZ fnlA fnlB fnlC wbfG
O119 rmlB rmlD rmlA wekD wekE wekF wekG rmlC wekH wekI manC manB wzx wekJ wzy wekK
O120 galF rmlB rmlD rmlA rmlC wzx wzy gnd
O121 wbqA wbqB rmlB rmlA vioA wbqC wbqD wzx wbqE wbqF wzy wbqG wbqH wbqI
O123 rmlB rmlA wzx vioA wfbA wfbB wfbC wfbD wfbE wzy wfbF fnlA rmlD fnlC wbwH wbuC
O124 wzx wfeO wfeP wfeQ wfeR wzy wfeS glf wfeT
O125ab* wzx wzy gmd fcl manC
O125ac* wzx wzy gmd fcl manC
O126 wzy wbgK wbgL wbsT wbsU gmd fcl manC manB wzx
O127 galF galE wbiN gmd fcl gmm manC manB wzx wbiO wzy wbiP wbiQ
O128 wbsH gmd fcl gmm manC manB wbsI wzx wbsJ wbsK wbsL wzy galE
O129 rmlB rmlD rmlA rmlC wzx rfbF rfbG wzy
O130 wzx wffF wffG wzy wffH' wffI wffJ gne
O131* nnaD nnaB nnaC phaR wzx wzy amsE
O132* rmlB rmlD rmlA rmlC wzx glf wbbL glgA lacA wzy
O133* rmlB rmlD rmlA rmlC wzx pglA wzy
O134* wfdR wzx wfgD_1 wzy wfgD_2
O135 rmlB rmlD rmlA rmlC wzx rfbF rfbG wzy
O136* pseB neuA pseG wcnT wzx wzy wfeD
O137* ispD wzy wzx wcnK wcnL wcnM wcnO
O138 rmlB rmlD rmlA rmlC wzx wfaF wfaG gna gne wfaH wzy
O139* rmlB rmlD rmlA rmlC wzy wzx pglA wbbL
O140* wzx glf rmlB rmlD rmlA rmlC wzy weoF wfgD
O141 rmlB rmlD rmlA rmlC wzx wfcA wfcB wzy wfcC wfcD manC manB
O142* rmlA rmlC wzx wzy pglJ galE
O143 wfdP wfdQ wfdR wfdS wzx wfdT wfdU wzy gne wfdV
O144* wzy pglH pimA gtf1 manC algC pssM wzx epsH
O145 wckD nanB nnaC nnaA wzx wzy wbuW wbuX wbuY wbuZ fnlA fnlB fnlC wbuB wbuC
O146 rmlB rmlD rmlA rmlC wbuD wzx wbuE wbwU wzy wbwV wbwW
O147 rmlB rmlD rmlA rmlC wzx wzy wfbY wfbZ
O148 rmlB rmlD rmlA rmlC wzx wzy wbbR wbbQ wbbG
O149
O150 rmlB rmlD rmlA rmlC wfgF wzx wfgG wfgH wfgI wfgK wzy
O151 wbhU wzx wbhV wzy wbhW wbhX wbhY wbhZ fnlA fnlB fnlC wbfG
O152 rmlB rmlD rmlA rmlC wfgB wzx wfgC wzy wfgD wfgE
O153* wbbD epsH wzy pglJ wzx mdoB
O154 galF rmlB rmlD rmlA rmlC wzx wzy gnd
O155 wclE wzy wclF wclG wzx wclD ugd gla wzz
O156* wzx wzm mshA wzy gtf1 gmd fcl gmm manC glmM
O157 wbdN wzy wbdO wzx per wbdP gmd fcl wbdQ manC manB wbdR
O158 gne rmlB rmlD rmlA rmlC wzx wzy wcmQ wcmR wcmS wcmT
O159 wzx wfgP wfgQ wzy wfgR gmd fcl gmm manC wfgS manB
O160* wzx epsJ epsH wzy sacB wbbD
O161 lea1 lea2 lea3 lea4 lea5 weiE lea6 lea7 wzx weiF wzy weiG
2 kb
dTDP glucose pathway genes
GDP rhamnose pathway genes
CDP-glycerol pathway genes
UDP-glucose pathway genes
Acetyl glycosamine
Glycosyl transferase
Epimerase
galF
gnd
wzx
wzy
wzt
wzm
Other

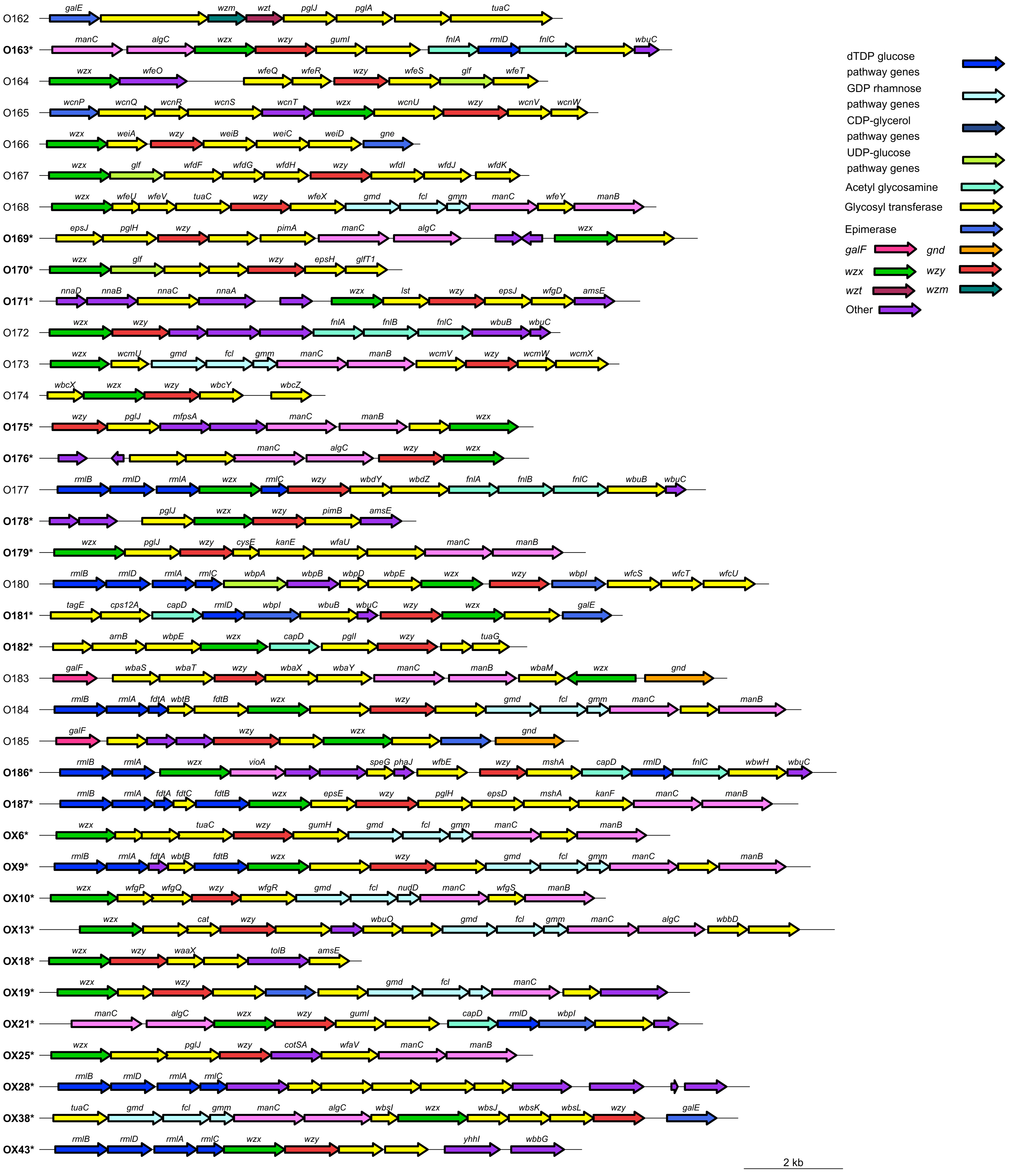
O162 galE wzm wzt pglJ pglA tuaC
O163* manC algC wzx wzy gumI fnlA rmlD fnlC wbuC
O164 wzx wfeO wfeQ wfeR wzy wfeS glf wfeT
O165 wcnP wcnQ wcnR wcnS wcnT wzx wcnU wzy wcnV wcnW
O166 wzx weiA wzy weiB weiC weiD gne
O167 wzx glf wfdF wfdG wfdH wzy wfdI wfdJ wfdK
O168 wzx wfeU wfeV tuaC wzy wfeX gmd fcl gmm manC wfeY manB
O169* epsJ pglH wzy pimA manC algC wzx
O170* wzx glf wzy epsH glfT1
O171* nnaD nnaB nnaC nnaA wzx lst wzy epsJ wfgD amsE
O172 wzx wzy fnlA fnlB fnlC wbuB wbuC
O173 wzx wcmU gmd fcl gmm manC manB wcmV wzy wcmW wcmX
O174 wbcX wzx wzy wbcY wbcZ
O175* wzy pglJ mfpsA manC manB wzx
O176* manC algC wzy wzx
O177 rmlB rmlD rmlA wzx rmlC wzy wbdY wbdZ fnlA fnlB fnlC wbuB wbuC
O178* pglJ wzx wzy pimB amsE
O179* wzx pglJ wzy cysE kanE wfaU manC manB
O180 rmlB rmlD rmlA rmlC wbpA wbpB wbpD wbpE wzx wzy wbpI wfcS wfcT wfcU
O181* tagE cps12A capD rmlD wbpI wbuB wbuC wzy wzx galE
O182* arnB wbpE wzx capD pglI wzy tuaG
O183 galF wbaS wbaT wzy wbaX wbaY manC manB wbaM wzx gnd
O184 rmlB rmlA fdtA wbtB fdtB wzx wzy gmd fcl gmm manC manB
O185 galF wzy wzx gnd
O186* rmlB rmlA wzx vioA speG phaJ wfbE wzy mshA capD rmlD fnlC wbwH wbuC
O187* rmlB rmlA fdtA fdtC fdtB wzx epsE wzy pglH epsD mshA kanF manC manB
OX6* wzx tuaC wzy gumH gmd fcl gmm manC manB
OX9* rmlB rmlA fdtA wbtB fdtB wzx wzy gmd fcl gmm manC manB
OX10* wzx wfgP wfgQ wzy wfgR gmd fcl nudD manC wfgS manB
OX13* wzx cat wzy wbuO gmd fcl gmm manC algC wbbD
OX18* wzx wzy waaX tolB amsE
OX19* wzx wzy gmd fcl manC
OX21* manC algC wzx wzy gumI capD rmlD wbpI
OX25* wzx pglJ wzy cotSA wfaV manC manB
OX28* rmlB rmlD rmlA rmlC
OX38* tuaC gmd fcl gmm manC algC wbsI wzx wbsJ wbsK wbsL wzy galE
OX43* rmlB rmlD rmlA rmlC wzx wzy yhhI wbbG
2 kb
dTDP glucose pathway genes
GDP rhamnose pathway genes
CDP-glycerol pathway genes
UDP-glucose pathway genes
Acetyl glycosamine
Glycosyl transferase
Epimerase
galF gnd
wzx wzy
wzt wzm
Other
